# ATM Regulates Differentiation of Myofibroblastic Cancer-Associated Fibroblasts and Can Be Targeted to Overcome Immunotherapy Resistance

**DOI:** 10.1158/0008-5472.CAN-22-0435

**Published:** 2022-11-10

**Authors:** Massimiliano Mellone, Klaudia Piotrowska, Giulia Venturi, Lija James, Aleksandra Bzura, Maria A. Lopez, Sonya James, Chuan Wang, Matthew J. Ellis, Christopher J. Hanley, Josephine F. Buckingham, Kerry L. Cox, Gareth Hughes, Viia Valge-Archer, Emma V. King, Stephen A. Beers, Vincent Jaquet, George D.D. Jones, Natalia Savelyeva, Emre Sayan, Jason L. Parsons, Stephen Durant, Gareth J. Thomas

**Affiliations:** 1 School of Cancer Sciences, Faculty of Medicine, University of Southampton, Southampton, United Kingdom.; 2 Department of Genetics and Genome Biology, Cancer Research Centre, University of Leicester, Leicester, United Kingdom.; 3 Department of Molecular and Clinical Cancer Medicine, Cancer Research Centre, University of Liverpool, Liverpool, United Kingdom.; 4 Bioscience, Oncology Innovative Medicines and Early Development (IMED) Biotech Unit, AstraZeneca, Cambridge, United Kingdom.; 5 Department of Pathology and Immunology, Centre Médical Universitaire, Genève, Switzerland.

## Abstract

Myofibroblastic cancer-associated fibroblast (myoCAF)–rich tumors generally contain few T cells and respond poorly to immune-checkpoint blockade. Although myoCAFs are associated with poor outcome in most solid tumors, the molecular mechanisms regulating myoCAF accumulation remain unclear, limiting the potential for therapeutic intervention. Here, we identify ataxia-telangiectasia mutated (ATM) as a central regulator of the myoCAF phenotype. Differentiating myofibroblasts *in vitro* and myoCAFs cultured *ex vivo* display activated ATM signaling, and targeting ATM genetically or pharmacologically could suppress and reverse differentiation. ATM activation was regulated by the reactive oxygen species–producing enzyme NOX4, both through DNA damage and increased oxidative stress. Targeting fibroblast ATM *in vivo* suppressed myoCAF-rich tumor growth, promoted intratumoral CD8 T-cell infiltration, and potentiated the response to anti–PD-1 blockade and antitumor vaccination. This work identifies a novel pathway regulating myoCAF differentiation and provides a rationale for using ATM inhibitors to overcome CAF-mediated immunotherapy resistance.

**Significance:**

ATM signaling supports the differentiation of myoCAFs to suppress T-cell infiltration and antitumor immunity, supporting the potential clinical use of ATM inhibitors in combination with checkpoint inhibition in myoCAF-rich, immune-cold tumors.

## Graphical Abstract

**Figure:**
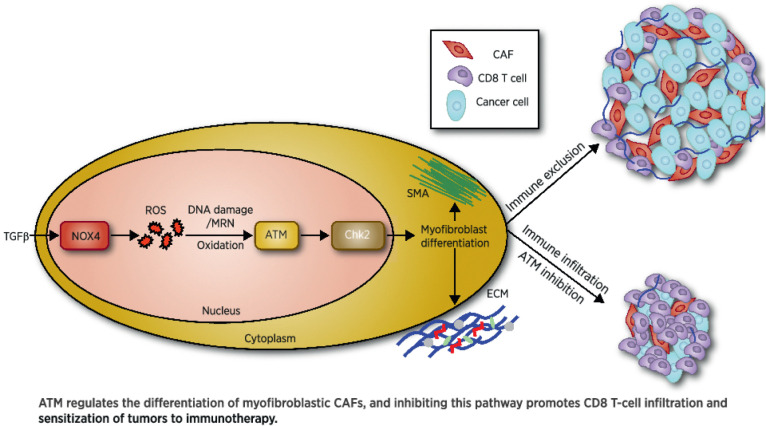

## Introduction

Immune-checkpoint blockade elicits durable antitumor clinical responses in an increasing number of malignancies. However, its success is limited to a fraction of cancer patients, highlighting the need to identify targetable resistance mechanisms to widen its clinical effectiveness (1). Analysis of human cancers and mouse models has shown that nonresponsiveness to immune-checkpoint blockade can result from limited T-cell infiltration, mediated by the cells of the tumor microenvironment (1).

Among the heterogeneous CAF population, a distinct myofibroblastic phenotype has been found in most types of solid tumors (2). Myofibroblastic CAF (myoCAF) are analogous to myofibroblasts found in wound-healing and tissue fibrosis. These contractile, secretory cells are regulated through TGFβ1 signaling, express α-smooth muscle actin (SMA), and secrete collagen-rich extracellular matrix (ECM; refs. 2, 3). Studies in multiple cancer types have shown that tumors with an SMA-positive, myoCAF-rich stroma are associated with poor prognosis (4–6) and that myoCAF contribute to many hallmarks of malignancy (7). Transcriptomic analyses of tumors that fail to respond to anti–PD-1/PD-L1 have identified upregulation of myoCAF genes (8–13), suggesting that myoCAF mediate resistance to checkpoint blockade. Consistent with this, myoCAF-rich cancers contain low levels of infiltrating T cells (8, 10, 14) and functional studies in murine models have confirmed that myoCAF promote resistance to different types of immunotherapy (8, 13, 14), in part by promoting T-cell exclusion from tumors. This has led to the emergence of myoCAF as potential therapeutic targets (15). However, a limited understanding of CAF heterogeneity and of the mechanisms regulating the accumulation of different CAF phenotypes has resulted in unsuccessful clinical trials, illustrating challenges to effectively target these cells (16–19).

TGFβ/SMAD signaling is recognized as the central pathway regulating myofibroblast/myoCAF differentiation, although other pathways also influence the myoCAF phenotype (3). We found previously that during TGFβ1-induced differentiation, myofibroblasts upregulate a number of genes associated with DNA repair, suggesting that the DNA damage response (DDR) pathway may be triggered during the process (20). The DDR is a complex cellular network of coordinated pathways that maintain genome integrity by detecting and repairing DNA lesions (21), orchestrated by DNA damage-sensing kinases ataxia-telangiectasia mutated (ATM), ataxia-telangiectasia mutated and Rad3-related (ATR), or DNA-dependent protein kinase (DNA-PKc; ref. 22). TGFβ/SMAD signaling has been reported to contribute to the cellular DDR induced by genotoxic insults (23, 24) or by radiation/stress-driven bystander effects (25–27). However, it remains to be determined if and how the TGFβ/SMAD pathway affects DNA repair during the acquisition of the myofibroblast/myoCAF phenotype.

The myofibroblast differentiation process is associated with the generation of intracellular reactive oxygen species (ROS), and we have previously described a role for the ROS-generating enzyme NADPH oxidase 4 (NOX4) in promoting and maintaining the myoCAF phenotype (28). Notably, the inactive noncovalent ATM homodimer is typically activated by the Mre11–Rad50–Nbs1 (MRN) complex in response to DNA double-strand breaks (DSB; refs. 29, 30) becoming a monomer through autophosphorylation. However, ATM can also be activated as a covalent dimer through oxidation and independent of DNA damage (31), suggesting a possible link between NOX4-generated oxidative stress, ATM activation, and myofibroblast differentiation.

Here we sought to investigate the role of the DDR signaling in regulating myoCAF phenotype and function using *in vitro/ex vivo* culture of myofibroblasts/myoCAF, analysis of human tumors, and CAF-rich murine tumor models that recapitulate the stromal morphology, immune microenvironment, and immunotherapy resistance found in CAF-rich human tumors (14).

## Materials and Methods

### Analysis of human tumors

Written informed consent was obtained from each patient (Rec No. 10/H0504/32, 09/H0504/66, and 14/SC/0186), and the National Research Ethics Service Committee South Central - Hampshire B approved the study protocol that followed the ethical guidelines of the 1975 Declaration of Helsinki. All tissue collection and storage were carried out by a human tissue authority (HTA)-licensed tissue bank. Automated immunostaining of full tissue sections was performed in a UKAS-accredited Cellular Pathology Laboratory in University Hospital Southampton. FFPE sections (4 μm) were mounted on Superfrost+ (Thermo Fisher Scientific) slides and preheated at 60°C for 30 minutes. All subsequent steps were completed using commercially available visualization systems [Envision FLEX (Dako)] and automated platforms [Dako PT Link (Dako); Autostainer Link48 (Dako)] optimized for use within a clinical diagnostic pathology laboratory. Deparaffinization, rehydration, and antigen retrieval were performed using Dako PT links as previously shown (14). The order of the primary antibody incubations was: (i) CD31 (1:5; DAB-Brown; IR61061-2 Dako); (ii) SMA (ready-to-use; AEC-RED; IR61161-2 Dako); (iii) pATM-ser1981 (1:200low AEC_RED; ab36810 Abcam); and (iv) CK (1:5; AE1/AE3; AEC_RED; IR05361-2, Dako).

Multiplex images were generated using a stain clearing–based method: nuclear counterstain (hematoxylin) and registration markers (CD31 using DAB) remain permanent, followed by sequential staining and clearing of a transient stain (SMA, pATM, and CK using AEC), as previously described (14).

SMA IHC (Sigma-Aldrich; A2547) on FFPE tissues was performed as previously described (14). Quantification of SMA staining in mouse tumors was carried out using color thresholding and deconvolution in Fiji (Supplementary File S1 for the macro in Fiji). At least three independent fields of view (FoV) of at least three tumors were randomly selected by a consultant pathologist (GJT) to generate a mean value per tumor representing the SMA-positive area percentage, which was compared between treatment groups.

CD8 and CD4 IHC was performed on frozen tissue embedded in OCT using [YTS169 (CD8; in house) and RM4-5 (CD4; BD; Fig. 6C and Supplementary Fig. S6E). Sections (8 μm) were fixed in 100% acetone for 10 minutes and stained as previously shown (14). CD8 and CD4 IHC on FFPE was performed using 98941S and 25229S, respectively (Cell Signaling Technology; Fig. 6A, G, K, O and Supplementary Figs. S6A–S6C, G and 7M and O) using the Leica Bond system according to the manufacturer's instructions (Protocol F mouse 4; HIER 20 MINUTES ER1; Temperature-Ambient; 1:100 dilution; 20 minutes of NGS blocking incubation). CD8 and CD4 T cells were counted from 10 randomly selected FoV from the tumor by a consultant pathologist (GJT).

### Cell culture

A human NSCLC cell line H441, HNSCC SCC25, murine cancer cell line TC-1 (lung), MC38 (colon), and human fetal foreskin fibroblasts HFFF2 were purchased from European Collection of Cell Cultures (Public Health England) or ATCC; HNSCC 5PT (32) were provided by I. Mackenzie (Queen Mary University of London, London, United Kingdom). HEK293T cells and IMR90 lung fibroblasts were provided by Jesus Gil (Imperial College, London, United Kingdom). SCC25 were grown in Ham's F12:DMEM (1:1 ratio) medium with 10% FBS (Calbiochem) and 292 μg/mL L-glutamine. 5PT were cultured in a keratinocyte growth medium (28). HEK293T, H441, MC38, HFFF2, IMR90, and primary fibroblasts were grown in DMEM supplemented with 10% FBS and 292 μg/mL L-glutamine. Primary fibroblasts were isolated as previously (20, 28) used at early passage (p1–10). Primary fibroblasts are listed in Supplementary File S2. C57BL/6 murine normal lung and colon fibroblasts (MLF and MCF, respectively) were grown at 3% oxygen (15). TC-1 were cultured in RPMI supplemented with 10% FBS and 292 μg/mL L-glutamine. All cell cultures were routinely tested for *Mycoplasma* contamination using Megamix-Blue according to the manufacturer's instructions (Clent Life Science—Microzone). All cells were cultured using standard cell culture plates/flasks (Corning). HFFF2 or IMR90 cells were transfected with 25 nmol/L On-Target pool siRNA (Thermo Scientific/Dharmacon) using Oligofectamine reagent (Life Technologies) as described (28). Stable knockdown was performed using retro/lentiviral mediated transduction of shRNA plasmids, as previously described (28) followed by cell selection using 1 μg/mL puromycin for 5 to 7 days. NOX4-inducible HEK293 cells (kindly provided by Vincent Jaquet) were treated with 1 μg/mL doxycycline (33). Wild-type and mutant (C2991L) ATM overexpression was carried out by transfecting HFFF2 with Viafect (Promega) according to the manufacturer's instructions and selected for 10 days with 4 mg/mL G418. p-Retrosuper-shATM (ATM#1:912) and pCDNA3.1-Flag-mut-ATM (C2991L; pTP1625 clone) were kindly provided by Yosef Shiloh and Tanya Paull (31), respectively. pLKO.1 murine shATM (TRCN0000012643) and pCDNA3.1-Flag-wt-ATM (31985) were purchased from Sigma and Addgene, respectively. 2 ng/mL TGFβ (TGFβ1; R&D Systems; for 3 days) was used to induce myofibroblast differentiation. 10 ng/mL IL1β (R&D Systems) for 72 hours was used to induce iCAF differentiation. Unless otherwise stated, 40 μmol/L GKT137831 (kindly provided by Genkyotex), 13.3 μmol/L KU55933 (ATM inhibitor), 2.5 μmol/L or 0.5 μmol/L KU60019 (ATM inhibitor), 1.5 μmol/L CCT241533 (CHK2 inhibitor), 40 μmol/L Mirin (Mre11 inhibitor), 0.5 or 2.5 μmol/L VE-821 (ATR inhibitor), 2 or 10 μmol/L NU-7441 (DNAPK inhibitor; all from Selleckchem), 1 μmol/L TGFβ-receptor I inhibitor (Calbiochem) and 0.5 μmol/L AZD0156 (ATM inhibitor provided by AstraZeneca) were added to the cells 1 hour prior to TGFβ1 treatment. All the inhibitors were resuspended in DMSO. To trigger DDR activation and/or cell stress, HFFF2 were either irradiated with 2 to 100 Gy using 350 Kv X-ray Irradiation System (Faxitron), or treated with 100 ng/mL neocarzinostatin for 2 hours (NCS; Sigma), with 2–10 mmol/L H_2_O_2_ (Fisher Scientific) or with 5 μg/mL cisplatin (Sandoz) for 30 minutes.

### Comet assay

HFFF2 cells (10 × 10^3^) were treated with TGFβ1 for 24 hours or x-irradiated (2 Gy) 30 minutes before harvesting, resuspended in 0.6% low melting point agarose (UltraPure LMP Agarose from Life Technologies), placed onto precoated slides with 1% normal melting point agarose, and coverslipped. Slides were placed on ice for 30 minutes to allow gels to solidify, coverslips were removed, and slides were placed in ice-cold lysis buffer (2.5 M NaCl, 0.1 M EDTA, 10 mmol/L Tris-HCl, 1% TritonX-100 at pH 10) in the dark, overnight. Next (with light protection), slides were washed for 10 minutes with ice-cold ddH_2_O and placed in ice-cold alkaline buffer (300 mmol/L NaOH, 1 mmol/L disodium EDTA pH > 13) for 20 minutes, and electrophoresed for 20 minutes at 30 v/300 mA protected from light. Following electrophoresis, slides were incubated with neutralization buffer (0.4M Tris-HCl, pH 7.5) for 20 minutes, washed twice for 10 minutes with ddH_2_O, and placed at 37°C overnight to dry. Slides were rehydrated with ddH_2_O for 30 minutes at room temperature, stained with propidium iodide (PI, 2.5 μg/mL) for a further 20 minutes, washed with fresh ddH_2_O for 20 minutes and placed at 37°C to dry before the comet scoring using an Olympus BH-2-RFL-T2 fluorescence microscope (Komet Analysis software version 5.5; Andor Technology). The percentage of DNA in the tail of the comet resulting from total DNA breaks (single- and double-strand breaks) was calculated for each cell. Six independent experiments were carried out with 50 cells/comets analyzed per replicate.

### Invasion assays

CAF, HFFF2, or shCTR/shATM HFFF2 populations were treated with 13.3 μmol/L KU55933 ± TGFβ1 for 7 days (treated days 1 and 4). Cells were washed twice with PBS, and serum-free DMEM was added for 72 hours. Fibroblast-conditioned medium was collected, centrifuged, filtered (0.2 μm), normalized according to cell number, and used as a chemoattractant in the lower chamber of Transwell invasion assays as previously (4, 20). Two-way ANOVA was used for statistical analysis.

### Fluorescence microscopy

5 × 10^3^ HFFF2 were plated on permanox chamber slides (Thermo Scientific) and treated as described in the figure legends. Immunostaining was performed as previously (28). DAPI was used as a nuclear counterstain, and coverslips were mounted using a fluorescent mounting solution (Dako). Images were taken using an Olympus IX81 fluorescence microscope and the Xcellence program. DNA-damage foci were quantified from at least 8 nonoverlapping FoV with ∼300 cells in total per condition. Kruskal–Wallis statistical test was used to indicate the presence of a different distribution per time point in each group of foci. For collagen 1A1 deposition, 5 × 10^3^ HFFF2 or MLF were seeded into chamber slides and treated with TGFβ1 ± inhibitors for a week (treated days 1 and 4). Decellularization was performed using 0.25M NH_4_OH in 50 mmol/L Tris at 37°C for 30 minutes and slides fixed with ice-cold 100% methanol at −20°C followed by a blocking step. The remainder was performed as previously described (28). Antibodies used for immunofluorescence are listed in Supplementary File 2. SMA and collagen 1A1 quantification was performed using Fiji (macro to calculate the image mean can be found in Supplementary File S1). High-resolution imaging of mouse tumors was carried out using an Axioscan.Z1 scanner (Zeiss). Immunocytochemistry for NOX4 was performed using antibodies from Novus Biologicals and Abcam with similar results (see Supplementary File S2 for antibody details).

### Viability, proliferation, and toxicity assays

To assess cell viability and proliferation, HFFF2s were plated in 96-well plates (5 × 10^3^ cells/well) and treated the following day with inhibitors ±TGFβ1. After 3 days, [H_3_]-thymidine was added to the medium for 16 hours, and radioactivity was measured using TopCount NXT (Packard Bioscience). MTT assay was carried out using CellTiter 96 AQueous Nonradioactive Cell Proliferation Assay (Promega) according to the manufacturer's instructions. The absorbance was read at 490 nm using a Victor plate reader (Perkin Elmer). To monitor cell death, HFFF2s were plated in 6-well plates (200 × 10^3^ cells/well) and treated the following day with inhibitors ± TGFβ1. After 3 days, cells were detached, resuspended in 300 μL of FACs buffer containing 1.7 ng/μL PI (Thermo Fisher) and analyzed using a Bd FACSCanto Flow Cytometer (BD Biosciences). Cells were less than 90% confluent at the end of each experiment. Cells were treated in the final 30 minutes with 10 mmol/L H_2_O_2_ (for PI and thymidine assay) or 5 μg/mL cisplatin (for MTT assay) as positive controls.

### Senescence and ROS assay

HFFF2s were plated in 24-well plates (1 × 10^3^ or 5 × 10^3^ cells/well) and treated with TGFβ1 the following day (and then every 3 days). Senescence was quantified at days 5 and 12 from TGFβ1 treatment determining the percentage of SA-β-Gal-positive cells (Sigma) of the total number of cells counterstained with Dapi (Sigma) as previously described (20). At least 200 cells/well in at least three independent experiments were counted per sample using a CKX41 Olympus fluorescent microscope. Cells were less than 50% confluent at the end of the experiment to avoid SA-β-Gal false-positive cells due to cell contact. ROS assay was performed as previously (28).

### Western blotting

Cells were lysed in ice-cold RIPA buffer [1% NP40, 150 mmol/L NaCl, 25 mmol/L Tris-HCl pH 7.5, 0.1% SDS, 1% Na-deoxycholate, 1 mmol/L EGTA, 1 mmol/L EDTA, 1 mmol/L Na-orthovanadate, 10 mmol/L Na-fluoride, 2.5 mmol/L Na-pyrophosphate, 1 mmol/L β glycerophosphate + freshly added 1 mmol/L PMSF, 20 mmol/L N-ethylmaleimide (NEM), 10 ng/mL Microcystin-LR, 0.12 μmol/L okadaic acid and phosphatase and protease inhibitor cocktails; all from Sigma]. Protein extracts were then centrifuged, and supernatants were quantified using the Dc-Bio-Rad protein assay kit (Bio-Rad Laboratories) as previously described (28). For nuclear-cytoplasmic protein extraction, we used NE-PER Reagents according to the manufacturer's instructions (Thermo Fisher). Equal amounts of protein were electrophoresed either in reducing (+Dithiothreitol, DTT) or in nonreducing conditions in 3% to 8% or 4% to 12% SDS-PAGE gels and electro blotted as previously (28). To detect the ATM dimer, cells were detached, washed once with PBS, and then kept moving at room temperature for 10 minutes with PBS + 100 mmol/L NEM before nuclear extraction. HSC70 was used as a loading control. Bound antibodies were detected using a chemiluminescence system (Pierce), visualized at the Fluor-S Multi-imager (Bio-Rad), quantified using Fiji, and were plotted as ratios between a given protein and HSC70 unless otherwise stated. Antibodies are described in Supplementary File S2.

### Quantitative real-time PCR

For quantitative real-time PCR (qRT-PCR), RNA was extracted and retrotranscribed as described previously (14), and qRT-PCR was performed as previously (28). The mRNA expression levels were analyzed using the ΔΔC_t_ relative quantitation method. GAPDH or HPRT were used as human or mouse housekeeping genes, respectively. Gene-specific primers were designed using http://www.ncbi.nlm.nih.gov/tools/primer-blast/. Primer sequences are given in Supplementary File S2.

### Collagen gel contraction assays

Fibroblasts (5 × 10^5^) were added to 1 mL of ice-cold 3 mg/mL type 1 collagen (Millipore) gels containing 10% FBS DMEM as described previously (21). Gels were incubated at 37C for 1 hour to polymerize. After adding 1 mL of 10% FBS DMEM, gels were detached using a spatula and photographed after 24-hour incubation. The gel area was measured using Fiji.

### *In vivo* mouse experiments

All mouse experiments were performed according to national guidelines and were approved by the authors' institutional review board and the UK Home Office. Xenograft model: 6.7 × 10^5^ 5PT cells ± 2 × 10^6^ HFFF2 cells were resuspended in 100 μL PBS and injected s.c. in the flank of partially immunocompromised, male RAG1^−/−^ C57BL/6 mice (3–5 months old). Isograft models: 0.5 × 10^5^ TC-1 cells ± 3 × 10^5^ TGFβ1-treated MLF (myoMLF) or 1 × 10^5^ MC38 cells ± 5 × 10^5^ TGFβ1-treated murine colon fibroblasts (myoMCF) were resuspended in 100 μL PBS and injected s.c. in the flank of C57/BL6 female mice (6–8 weeks old). Five to eight animals for both models were used per group. Tumor size was measured over time using an electronic caliper and calculated using the formula 4π/3 × *r*3 [radius (*r*) was calculated from the average diameter, measured as the tumor width and length]. AZD0156 (ATMin; provided by AstraZeneca) was tested only on TC-1 and MC38 models (5PT cells were generated using prolonged cisplatin treatment and potentially have an altered DDR pathway (32)). Vaccination with DNA vaccine encoding tetanus fragment C domain 1 (Dom) fused to the immunodominant CD8 epitope of HPV E7 RAHYNIVTF (RAH, E749-57) p.Dom-RAH (34) was administered once via intramuscular injection (i.m.) when tumors were palpable (days 8–13; 20 μg of DNA in 100 μL PBS). P.Dom without the epitope served as a control. αPD-1 antibody (300 μg; Bio X Cell; RMP1-14) or IgG2a isotype control was given via intraperitoneal (i.p.) injection when tumors were palpable every other day, totaling 3 doses starting from days 9 to 14 from the tumor challenge. When tumors were palpable (at days 8–21 for TC-1 model and at days 8–9 for the MC38 model), mice were treated with oral gavage daily with either vehicle or 20 mg/Kg AZD0156 (ATMin) until the end of the experiment (as per the manufacturer's instructions).

For TC-1 survival analysis, mice were injected with RAH/CTR vaccines on day 14; AZD0156 was administered daily from days 15 to 47 and then twice a week until the end of the experiment (day 82). For MC38 survival analysis, αPD-1 was administered on days 14, 16, and 18; AZD0156 was given daily from days 9 to 28 and then twice weekly until the end of the experiment (day 47).

To test the effect of retreatment following relapse (Supplementary Fig. S7E–S7O), mice were injected with MC38 + myoMCF and treated when tumors were palpable with αPD-1 (at day 12, 14, and 16) ±AZD0156 (20 mg/kg; days 6–16; daily; first treatment). At relapse (tumor size ≥ 500 mm^3^), a second treatment with αPD-1 (2 doses; spaced at 48 hours when tumor size ≥ 700/800 mm^3^) ±AZD0156 (tumor size ≥ 500 mm^3^; 20 mg/kg; daily treatment for 10 days) was administered. Tumors were collected for IHC after the first treatment, at relapse, and when they reached the tumor size limit (1750 mm^3^). Mice whose tumors were used for IHC, whose size was ≥700/800 mm^3^, or had regressed completely after the first treatment (2/17 after ATMin + αPD-1 and 0/10 after αPD-1) were not included in the second treatment.

For tumor growth curves, statistical testing was performed on the mean area under the curve (AUC) using a two-way ANOVA. Following euthanasia, tumors were excised and either fixed in 10% formalin and embedded in paraffin or directly embedded in OCT matrix (Thermo Scientific) for IHC processing or disaggregated for FACS staining as previously (EF506 viability dye was used for live/dead cell staining; see Supplementary File S2 for antibodies; ref. 14).

### Bioinformatic analysis

Laser capture microdissected (LCMD) stroma from Gene Expression Omnibus (GEO) data sets [GSE45001 (liver), GSE35602 (colon), GSE19632 (esophagus), and GSE40595 (ovary)] were confirmed to be median normalized, preranked based on fold change (*P*_adj_ ≤ 0.05) between tumor versus normal stroma, and gene set enrichment analysis (GSEA) was run against DDR/ATM, TGFβ, and myoCAF (myCAF) genesets (https://www.gsea-msigdb.org/gsea/msigdb/; Supplementary File 3; refs.^12, 20, 28^). Normalized enrichment score (NES) ≥ 0 indicates a positive correlation and B&H *P*_adj_ (FDR *q*) ≤0.05 was considered significant. The DDR_TGFB_signature gene set was created using the Leading-Edge tool in GSEA from a previously published data set (TGF_UP_MELLONE_AGING; ref. 20). Briefly, TGFβ DEGs were ranked based on fold change, and GSEA was performed on genesets containing reference to DNA damage repair in their description and also on H_G2M_checkpoint and H_DNA_repair genesets. Genesets with significant enrichment (FDR *q* ≤ 0.25, according to GSEA guidelines) were selected manually excluding the ones not strictly correlated with DNA damage and repair (the full list is provided in Supplementary File S4). The list of the leading-edge genes in these genesets was made nonredundant (Supplementary File S5) and used to analyze the DDR enrichment in the GEO data sets mentioned above.

### Data availability

The data generated in this study are available within the article and its supplementary data files. Expression profile data analyzed in this study were obtained from GEO at GSE45001, GSE35602, GSE19632, and GSE40595

### Statistical analysis

All experiments were performed at least twice and data are expressed as the mean ± standard error unless otherwise stated (SD = standard deviation). Data are presented as a group with the number of biological replicates shown in the figure and the number of technical replicates described in the figure legends (*n*_br_ or *n*_tr_, respectively) and summarized by mean ± 95% confidence intervals in all figures. The appropriate use of parametric versus nonparametric tests was determined using the D'Agostino and Pearson omnibus normality test, and where sample sizes were too small for this test, a normal distribution was assumed. The Grubbs test was used to identify outliers. Where data did not follow a normal distribution pattern, Kruskal–Wallis, Mantel–Cox (log-rank) or Spearman tests were used. For normal distributions, two-way ANOVA, or homo/paired/heteroscedastic Student *t* test were used as described in the figure legends. Statistical tests were carried out using GraphPad Prism v. 6-8, and were two-sided; a *P* ≤ 0.05 was considered significant (ns, nonsignificant; *, P ≤ 0.05; **, P ≤ 0.01; ***, P ≤ 0.001; ****, P ≤ 0.0001).

## Results

### ATM activation during myofibroblast differentiation

**Figure 1.**
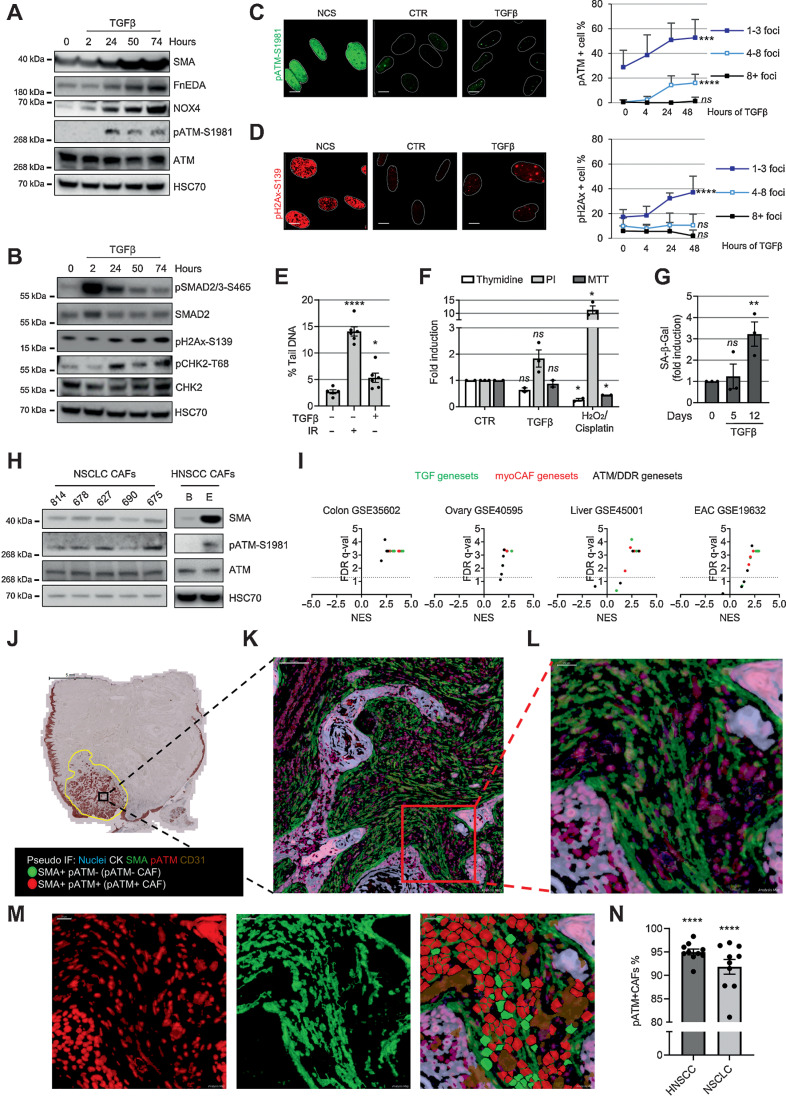
ATM activation in myofibroblasts. **A** and **B,** Western blotting of HFFF2 treated with TGFβ1 over time. **C** and **D,** Representative immunofluorescence staining of pATM- (**C**) and pH2AX-positive (**D**) foci, and quantification of HFFF2 treated over time with TGFβ1 or NCS (positive control); nuclei are outlined by dotted white lines based on DAPI nuclear counterstaining (not shown). Scale bar, 10 μm. Foci counts are expressed as percentage of total cell number per FoV (FoV = *n*_tr_ ≥ 8); SD and Kruskal–Wallis test are shown. **E,** Alkaline comet assay of HFFF2 treated with TGFβ1 for 24 hours or irradiated with 2 Gy (as positive control; *n*_tr_ = 50; homoscedastic). Student *t* test refers to the control. **F,** Analysis of viability, proliferation, and cell death monitored by MTT, thymidine incorporation, and PI staining, respectively. HFFF2 were treated for 3 days with TGFβ1 or with H_2_O_2_ or cisplatin as positive controls (*n*_tr_ = 2–3). **G,** Senescence assay of HFFF2 treated with TGFβ1 over time. The percentage of SA-β-Gal–positive cells is expressed as fold induction compared with untreated control; FoV = *n*_tr_ = 10. **H,** Western blotting of NSCLC and HNSCC CAF. **I,** Volcano plots with FDR *q* (significance) and NES (correlation) of GSEA performed on the indicated data sets from LCMD tumor versus normal stroma. The dotted line drawn at 1.3 of −log_10_ FDR *q* axis indicates FDR *q* = 0.05. **J–N,** Representative image of HNSCC MxIHC; brightfield image of cytokeratin staining. Scale bar, 5 mm (**J**); scale bar, pseudocoloered images, 100 μm (**K**); scale bar, 20 μm (**L** and **M**). Single-stained and merged pseudocolored images with the cell regions used for the quantification are highlighted in red or green for pATM or SMA positivity, respectively (subtracted CD31 staining is shown in brown); quantification of pATM positivity in SMA^+^ cells (negative for CD31 and CK) in 10 HNSCC and 10 NSCLC cases (**N**) with significance calculated comparing pATM^+^/SMA^+^ vs. pATM^−^/SMA^+^ CAFs. Paired Student *t* test is used in the figure and refers to control unless otherwise stated. ns, nonsignificant; *, *P* ≤ 0.05; **, *P* ≤ 0.01; ****, *P* ≤ 0.0001.

To investigate the DDR pathway during myofibroblast differentiation, we treated fibroblasts with TGFβ1 and examined the activation of DDR kinases over time alongside myofibroblast markers [SMA, fibronectin EDA (FnEDA)]. We found activation of ATM (but not ATR or DNA-PKcs) during differentiation (Fig. 1A and Supplementary Fig. S1A–S1E), whereas some ATM targets (H2AX and CHK2) were activated following TGFβ1 treatment, others (p53 and KAP1) were not (Fig. 1B and Supplementary Fig. S1E), indicating that only a subset of ATM targets typically phosphorylated during genotoxic stress are activated during myofibroblast differentiation. We also found that TGFβ1 treatment resulted in increased (albeit low) levels of pATM/pH2AX-positive nuclear foci and DNA breaks (Fig. 1C–E). This low level of DNA damage was associated with nonsignificant trends for reduced DNA replication and increased cell death; there were no changes in cell viability (monitored 3 days after TGFβ1 treatment) or senescence (5 days; Fig. 1F and G). However, as previously shown (35), continuous TGFβ1 administration resulted in increased senescence levels over time (% range SA-β-Gal–positive cells at 12 days = 15.8–23.3; Fig. 1g), suggesting overall that accumulation of DNA damage induced by TGFβ1 may ultimately promote cell senescence in a proportion of myofibroblasts.

We next examined CAF cultured *ex vivo* and human tumors *in vivo* for evidence of ATM activation. CAF from non–small cell lung cancer (NSCLC) and head and neck (HNSCC) showed ATM activation *in vitro*, which correlated with SMA expression (Spearman correlation = 0.96, *P* = 0.0028; Fig. 1H). GSEAs of microdissected tumor stroma data sets showed that myoCAF stroma of esophageal, ovarian, colorectal, and liver cancers are enriched for genes associated with the ATM/DDR pathway (Fig. 1I and Supplementary File 3). Multiplex immunochemistry (MxIHC) confirmed that the majority of SMA-positive CAF in NSCLC and HNSCC express pATM (Fig. 1J–N and Supplementary Fig. S1F–S1I), indicating, overall, that myoCAF *in vitro* and *in vivo* display activated ATM signaling.

### Targeting ATM inhibits myofibroblast differentiation

**Figure 2.**
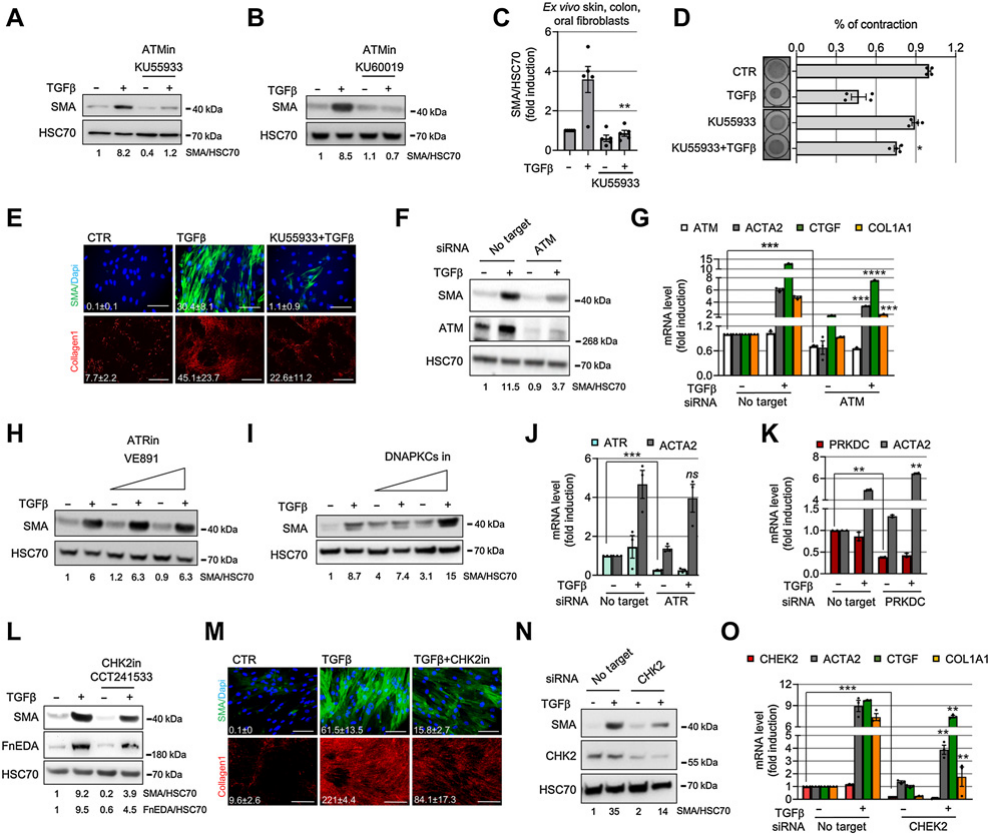
ATM inhibition suppresses myofibroblast differentiation. **A** and **B,** Western blotting and its quantification of HFFF2 treated for 72 hours with TGFβ1 ± ATM inhibitors (13.3 μmol/L KU55933, **A**; 2.5 μmol/L KU60019, **B**). **C,** Western blotting quantification of SMA expression in primary fibroblasts isolated *ex vivo* from colon (*n* = 2), skin (*n* = 1), and oral (*n* = 2) tissues from healthy donors. Fibroblasts were treated as in **A** (see also Supplementary Fig. S2C–G). **D,** Collagen gel contraction assay and measurement of gel area. HFFF2 or normal primary oral fibroblasts were treated as in **A**. Left, representative gel images (*n*_tr_ = 2). **E,** Representative immunofluorescence staining of SMA-positive stress fibers or collagen 1 deposition in HFFF2 treated with TGFβ1 ± ATM inhibitor as above for 3 (SMA) or 7 (collagen 1) days; relative quantification of the mean (FoV for both SMA and collagen 1 = 10). Scale bars for SMA, 100 μm and for collagen 1, 500 μm. **F** and **G,** Western blotting and quantification (**F**) and qRT-PCR (*n*_tr_ = 3; **G**) of HFFF2 transfected as indicated and treated with TGFβ1 for 72 hours (*ACTA2* = SMA gene). **H** and **I,** Western blotting and quantification of HFFF2 fibroblast treated for 72 hours with TGFβ1 ± ATR inhibitor (VE891; 0.5 μmol/L and 2.5 μmol/L; **H**) or ± DNA-PKcs inhibitor (2 μmol/L and 10 μmol/L; **I**) for 72 hours. **J** and **K,** qRT-PCR of HFFF2 transfected as indicated and treated with TGFβ1 for 72 hours (*n*_tr_ = 3; *PRKDC*, DNA-PKcs). **L,** Western blotting and quantification of HFFF2 treated for 72 hours with TGFβ1 ± CHK2 inhibitor (1.5 μmol/L CCT241533). **M,** Representative immunofluorescence staining of SMA and collagen 1 in HFFF2 treated with TGFβ1 ± CHK2 inhibitor (quantified as in **E**). **N** and **O,** Western blotting and quantification (**N**) and qRT-PCR (*n*_tr_ = 3; **O**) of HFFF2 transfected as indicated and treated with TGFβ1 for 72 hours. Heteroscedastic Student *t* test is used throughout the figure and is relative to TGFβ1-treated samples unless otherwise highlighted. *, *P* ≤ 0.05; **, *P* ≤ 0.01; ***, *P* ≤ 0.001; ****, *P* ≤ 0.0001.

To determine if ATM plays a functional role in myofibroblast differentiation, we cotreated fibroblasts with TGFβ1 and an ATM inhibitor (KU55933). ATM inhibition suppressed differentiation, inhibiting expression of SMA (Fig. 2A–C and Supplementary Fig. S2A–S2H), contraction of collagen gels, SMA-positive stress fiber formation, and collagen1 deposition (Fig. 2D and E; Supplementary Fig. S2I). We validated these findings using a second ATM inhibitor (KU60019) and ATM si/shRNA knockdown, which resulted in a similar reduction of SMA (*ACTA2*; Fig. 2B, F, and G; Supplementary Fig. S2J–S2L) and expression of ECM genes (*COL1A1* and *CTGF* genes; Fig. 2G). Overexpression of a dominant-negative Mut-ATM (C2991L; refs. 31, 36) also suppressed expression of SMA and ECM genes (Supplementary Fig. S2M–S2O). Targeting ATR or DNAPK did not reduce SMA expression (Fig. 2H–K), consistent with their lack of activation during myofibroblast differentiation (Supplementary Fig. S1E).

Recent single-cell transcriptomic studies have identified an inflammatory CAF (iCAF) subpopulation in several cancer types and described CAF plasticity, whereby cells in culture can be skewed between myoCAF and iCAF phenotypes by modulating TGFβ1 and IL1 signaling, respectively (12, 37). We, therefore, compared the effect of ATM inhibition on the expression of myoCAF and iCAF genes. KU55933 suppressed TGFβ1 induction of myoCAF genes (*COL11A1, ASPN*, and *ELN*) and significantly upregulated IL1β induction of most iCAF genes (*CCL2, LIF, IL6*, and *CXCL1*; Supplementary Fig. S2P–S2S). This hints at a dual role for ATM in determining fibroblast phenotype: as promoter of myofibroblast differentiation and as inhibitor of inflammatory CAF phenotype.

We next investigated whether CHK2, a downstream target of ATM, is also involved in myofibroblast differentiation. For this, we used a CHK2-specific inhibitor CCT241533 (38) and found, similar to ATM inhibition, that it suppresses TGFβ1-dependent SMA and FnEDA expression, SMA-positive stress fiber formation and collagen deposition (Fig. 2L and M and Supplementary Fig. S2T and S2U). siRNA targeting CHK2 produced a similar effect, decreasing the expression of SMA (*ACTA2*), *COL1A1*, and *CTGF* (Fig. 2N and O). Overall, these data indicate that ATM and its downstream signaling are central to the development of the myofibroblast phenotype.

### ATM maintains the established myoCAF phenotype

**Figure 3.**
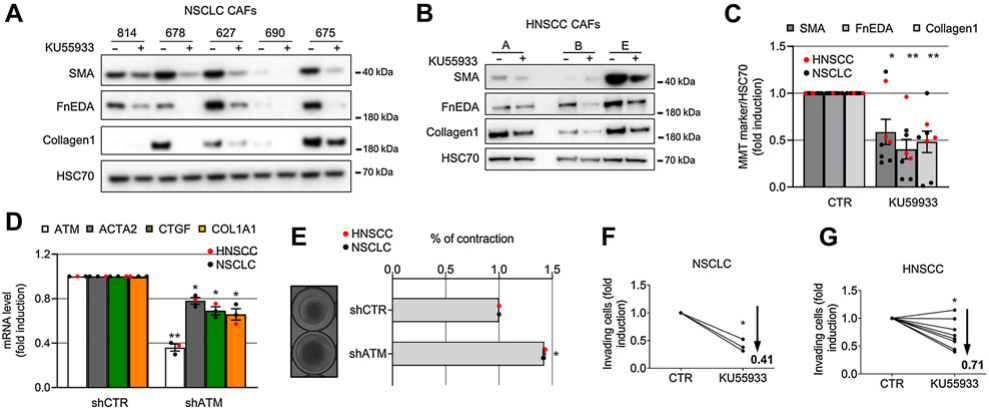
ATM inhibition reverses the myofibroblast CAF phenotype and inhibits function. **A–C,** Western blotting of HNSCC (**A**) and NSCLC (**B**) CAF treated with KU55933 for 7 days and their quantification (**C**). **D,** qRT-PCR of CAF (NSCLC, *n* = 1; HNSCC, *n* = 2) stably expressing either shCTR or shATM (*n*_tr_ = 3). **E,** Collagen gel contraction assay and measurement of gel area of shCTR or shATM HNSCC and NSCLC CAF (left, representative gel images; *n*_tr_ = 2). **F** and **G,** Transwell invasion assays of H441 (**F**) or 5PT (**G**) cells toward CAF-conditioned media generated from NSCLC (*n*_br_ = 3; *n*_tr_ = 3–5; **F**) and HNSCC (*n*_br_ = 8; *n*_tr_ = 2–4; **G**) treated as in **A** (means are shown and two-way ANOVA is used and refers to the control). Heteroscedastic Student *t* test is shown throughout the figure and refers to the control unless otherwise stated. *, *P* ≤ 0.05; **, *P* ≤ 0.01.

Continued ATM activation in differentiated myoCAF *in vitro* and *in vivo* (Fig. 1H–N) suggests that it may play a role in maintaining the established myoCAF phenotype. We, therefore, examined the effect of ATM targeting on HNSCC and NSCLC CAF cultured *ex vivo.* We found that inhibiting ATM using KU55933 or shRNA knockdown, reverted CAF to a more quiescent phenotype, downregulating the expression of myofibroblast markers (SMA, collagen I, fibronectin; Fig. 3A–D), suppressing contractility (Fig. 3E) and inhibiting myoCAF-dependent cancer cell invasion (Fig. 3F and G). Similarly, ATM inhibition or knockdown suppressed the invasion-promoting effects of myofibroblasts generated through TGFβ1 treatment (Supplementary Fig. S3A–S3C). Together, these data highlight that ATM can be targeted to both inhibit myoCAF differentiation and also “normalize” established myoCAF.

### TGF**β**1 activates ATM via NOX4

**Figure 4.**
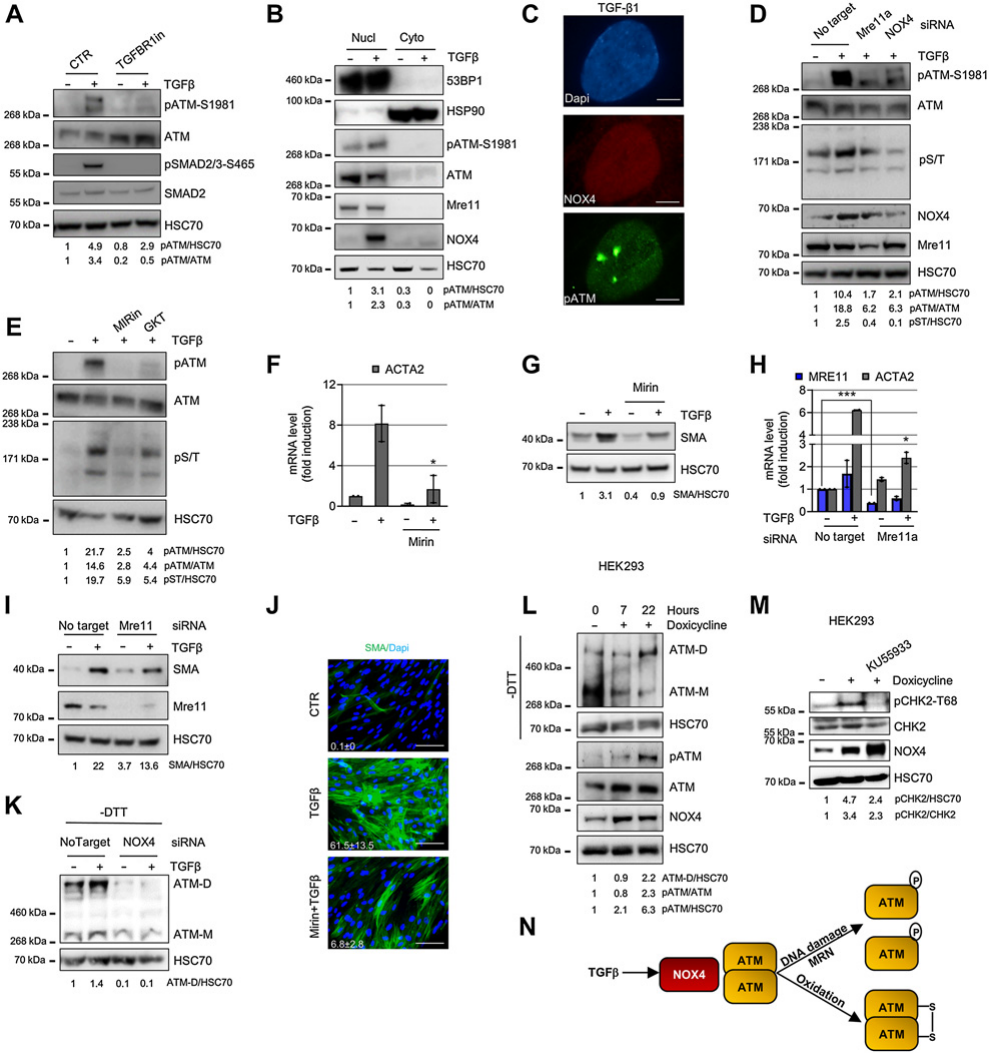
TGFβ activates ATM via NOX4-driven DNA damage/MRN complex and oxidation. **A–E,** HFFF2 were treated with TGFβ1 for 24 hours. **A,** Western blotting/quantification of HFFF2 treated with TGFβR1 inhibitor. **B**, Western blotting/quantification of nucleus/cytoplasm extracts using HSP90 and 53BP1 as cytoplasmic and nuclear marker, respectively. **C,** Representative immunofluorescence staining for NOX4 and pATM. Scale bar, 5 μm. **D,** Western blotting/quantification of HFFF2 transfected as indicated. **E,** Western blotting/quantification of HFFF2 treated with inhibitors of MRE complex inhibitor (Mirin, 40 μmol/L) or NOX4 (GKT137831; 40 μmol/L). **F** and **G**, qRT-PCR (*n*_tr_ = 3; **F**) and Western blotting/quantification (**G**) of HFFF2-treated TGFβ1 for 72 hours ± 40 μmol/L Mirin. **H** and **I,** qRT-PCR (*n*_tr_ = 3; **H**) and Western blotting/quantification (**I**) of IMR90 fibroblasts transfected as indicated and treated with TGFβ1 for 72 hours. **J,** Representative immunofluorescent staining of SMA-positive stress fibers and relative quantification of the mean in HFFF2 treated with TGFβ1 for 72 hours ± Mirin (40 μmol/L; scale bar, 100 μm; FoV = 10). **K,** Western blotting/quantification of HFFF2 transfected as indicated and treated with TGFβ1 for 48 hours; gel run in nonreducing -dithiothreitol (DTT) conditions for ATM dimer (ATM-D). **L,** Western blotting/quantification (±DTT) of NOX4-inducible HEK293 cells treated with doxycycline over time. **M,** Western blotting/quantification of NOX4-inducible HEK293 cells treated with doxycycline ± KU55933 for 22 hours. **N,** Schematic diagram of the main findings in the figure. Heteroscedastic Student *t* test is used in the figure and refers to the TGFβ1-treated samples unless otherwise highlighted. *, *P* ≤ 0.05; ***, *P* ≤ 0.001.

We next investigated the molecular mechanisms underlying TGFβ1-dependent activation of ATM. First, we inhibited TGFβ-receptor I phosphorylation of SMAD2/3 and confirmed that this abolished ATM activation in TGFβ1-treated fibroblasts (Fig. 4A). NOX4 is a SMAD downstream target (39), which we have shown previously regulates myoCAF differentiation (28); it has been reported that the ROS generated by this enzyme can cause DNA damage, suggesting a possible nuclear localization for NOX4 (27). We found that ATM phosphorylation and NOX4 expression increased with similar kinetics during the myofibroblast differentiation process (Fig. 1A and Supplementary Fig. S1A–S1C) and both localized to the cell nucleus (Fig. 4B and Supplementary Fig. S4A–C) but did not specifically colocalize (Fig. 4C). Targeting NOX4 using siRNA or a NOX4 inhibitor (GKT137831; ref. 28) suppressed ROS production (Supplementary Fig. S4D) and inhibited ATM phosphorylation, as well as its downstream kinase activity (Fig. 4D and E). DNA damage monitored by pH2AX protein expression was also decreased (Supplementary Fig. S4E and S4F). Because ATM is classically activated through DNA damage recognition by the MRN complex (29, 30), we examined whether Mre11 was directly involved in TGFβ1-dependent activation of ATM. Targeting Mre11 using siRNA or using a specific Mre11 inhibitor (Mirin) inhibited TGFβ1-driven ATM activation/activity similar to NOX4 inhibition (Fig. 4D and E). Consistent with this, Mre11 inhibition suppressed myofibroblast differentiation (Fig. 4F–J and Supplementary Fig. S4G and S4H).

Given that ATM can also be activated as a covalent dimer by oxidative stress (31), we next examined whether NOX4-driven ROS promotes ATM dimerization. First, we confirmed ATM dimer (ATM-D) formation by oxidation (H_2_O_2_; Supplementary Fig. S4I), before examining the effect of NOX4 inhibition. We found that NOX4 targeting resulted in a marked reduction in basal and TGFβ1-induced levels of the ATM dimer (Fig. 4K and Supplementary Fig. S4L). Targeting Mre11 did not affect ATM dimerization (Supplementary Fig. S4J and S4K), consistent with the MRN complex being involved in TGFβ1 activation of the monomer. Targeting NOX4 using GKT also inhibited ATM activation and dimerization in HNSCC and NSCLC CAF (Supplementary Fig. S4M–S4Q). To confirm the role of NOX4 in ATM activation, we overexpressed NOX4 in HEK293 cells (33). This induced ATM activation, dimerization, and activity (the latter monitored by CHK2 phosphorylation) without the requirement for TGFβ1 stimulation (Fig. 4L and M), suggesting that NOX4 activation and oxidation of ATM are not cell type specific. These data, summarized in the schematic in Fig. 4N, show that activation of ATM during myofibroblast differentiation is modulated by NOX4 both through DNA damage/MRN complex and by direct oxidation of ATM.

### ATM targeting in myofibroblasts reduces tumor growth

**Figure 5.**
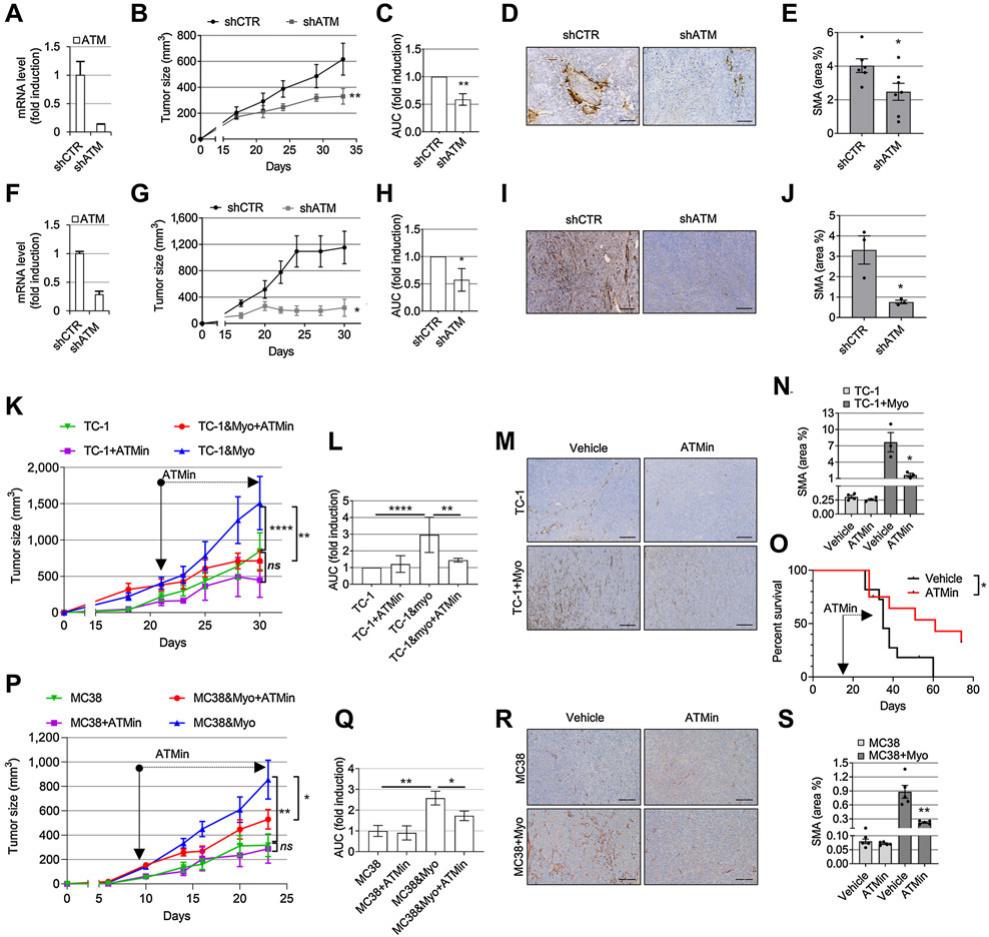
Targeting ATM in myofibroblasts reduces their intratumoral accumulation and slows tumor growth. **A** and **F,** qRT-PCR showing shRNA ATM knockdown in HFFF2 (**A**) or TGFβ1-treated MLF (myoMLF; **F**) prior to injection in mice (*n*_tr_ = 3; SD shown). **B, G, C,** and **H,** Tumor growth curves (**B** and **G**) and AUC histograms (**C** and **H**) following coinjection of tumor cells with shCTR or shATM fibroblasts (5PT cells + HFFF2, **B** and **C**; TC-1 cells + myoMLF, **G** and **H**). Data from single experiments are presented; mouse numbers = 3–8 (**B** and **G**). Two-way ANOVA is used for AUC analysis of three individual experiments for both 5PT (**C**) and TC-1 models (**H**). **D** and **I,** Representative SMA IHC from the experiments shown in **B** and **G**, respectively. **E** and **J**, Quantification of SMA staining (*n*_tr_ = FoV = 3) from the experiments shown in **B** (**E**) and **G** (**J**). **K, L,P,** and **Q**, Mice injected with either TC-1 ± myoMLF (**K** and **L**) or MC38 ± TGFβ1-treated MCF (myoMCF; **P** and **Q**) were treated with ATM inhibitor AZD0156 for the duration of the experiment (mouse number = 5–8); tumor growth curves (**K** and **P**); AUC analysis of two experiments relative to **K** (two-way ANOVA; **L**); AUC analysis of the single experiment shown in **P** (homoscedastic Student *t* test; **Q**). **M, N, R,** and **S,** Representative images and quantification of SMA IHC of mouse tumors in **K** and **P,** respectively (*n*_tr_ = FoV = 3). **O,** Overall survival of TC-1 + myoMLF mice treated daily with AZD0156 at days 15–28 (mouse number = 11–12; Mantel–Cox log-rank test is shown; see also Supplementary Fig. S5K and S5L). Homoscedastic Student *t* test is shown in the figure and refers to the control unless otherwise highlighted. Scale bars, 200 μm. ns, nonsignificant; *, *P* ≤ 0.05; **, *P* ≤ 0.01; ****, *P* ≤ 0.0001.

Next, we tested whether ATM targeting affects tumor growth *in vivo* using different murine tumor models (14, 28). First, we coinjected immunocompromised mice with 5PT cancer cells, a cell line that promotes myoCAF differentiation (28), with either control HFFF2 fibroblasts or HFFF2 with ATM shRNA knockdown (Fig. 5A). Stromal ATM targeting attenuated myoCAF accumulation and suppressed tumor growth (Fig. 5B–E). To investigate the role of fibroblast ATM targeting in immunocompetent mouse models, and to overcome the issue of low CAF content in commonly used syngeneic murine tumors, we used a syngeneic isograft lung cancer model as described previously (14), coinjecting TC-1 cancer cells with TGFβ1-treated MLF, which recapitulates the myoCAF-rich stroma of human tumors (Supplementary Fig. S5A–S5C). We found that myofibroblast ATM shRNA knockdown similarly suppressed intratumoral myoCAF accumulation and reduced tumor size in this model (Fig. 5F–J).

We then evaluated the effect of AZD0156, a clinically tested ATM-specific inhibitor (NCT02588105). We confirmed that AZD0156 suppressed myofibroblast differentiation *in vitro* (Supplementary Fig. S5D–G) and also reversed myoCAF differentiation; notably, this latter effect was maintained when AZD0156 treatment was discontinued (Supplementary Fig. S5H). A broad analysis of myoCAF, iCAF, and other immune genes showed that most myoCAF genes were significantly downregulated by ATM inhibition, whereas the effect on iCAF genes was gene specific and generally nonsignificant (Supplementary Fig. S5I and S5J). We next tested the effect of the inhibitor on mice injected with TC-1 cells ±TGFβ1-treated MLF (myoMLF). In mice bearing myoCAF-rich TC-1 tumors, AZD0156 suppressed myofibroblast accumulation, slowed tumor growth, and improved overall survival (Fig. 5K–O and Supplementary Fig. S5K–S5L). However, AZD0156 had minimal effect on the growth of control (myoCAF-low) tumors, showing that the effect of AZD0156 is specific to myoCAF in this model (Fig. 5K and L). Similar results were obtained using an isogenic colorectal cancer mouse model, coinjecting MC38 cancer cells with TGFβ-treated mouse colorectal fibroblasts (myoMCF; Fig. 5P–S). Together, these data show that ATM can be targeted to suppress intratumoral myoCAF accumulation, resulting in reduced tumor growth.

### ATM myoCAF targeting increases intratumoral CD8 T-cell infiltration and potentiates immunotherapy

**Figure 6.**
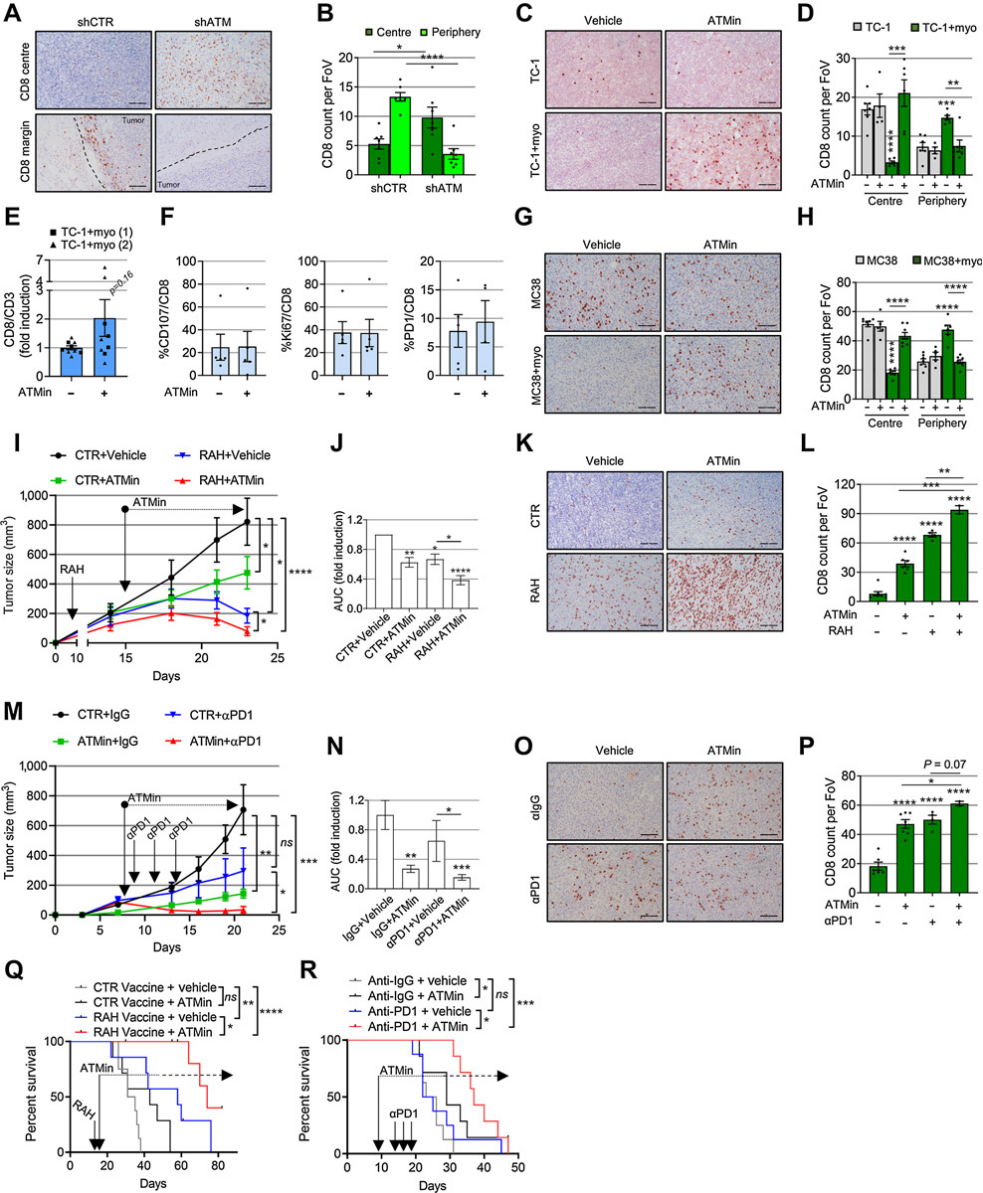
Targeting myofibroblast ATM promotes tumor CD8 T-cell infiltration and potentiates immunotherapy. **A** and **B,** Representative IHC staining (**A**) and relative quantification (**B**) of CD8 T cells in the core and periphery of TC-1 myo-rich tumors (described in Fig. 5F–J; *n*_tr_ = FoV = 10; dotted lines, tumor margins). **C** and **D,** Representative IHC staining (**C**) and relative quantification (**D**) of CD8 T cells in the core and periphery of tumors described in Fig. 5K (*n*_tr_ = FoV = 10). **E** and **F,** Flow cytometry analysis from TC-1 + myo-rich tumors described in Fig. 5K (**E** shows two experiments; two-way ANOVA was used). **G** and **H,** Representative IHC staining (**G**) and relative quantification (**H**) of CD8 T cells in the core and periphery of the tumors described in Fig. 5P (*n*_tr_ = FoV = 10). **I–L,** Mice were injected with TC-1 + myoMLF and treated with RAH vaccine±AZD0156. Control plasmid with vehicle was used as control. Tumor growth curves of a representative experiment (**I**; mouse number = 7–8; see also Supplementary Fig. S7A); two-way ANOVA is shown and refers to AUC analysis of three experiments (**J**). Representative IHC staining (**K**) and relative quantification (**L**) of CD8 T cells in the tumor core (*n*_tr_ = FoV = 10). **M–P,** Mice were injected with MC38 and myoMCF and treated with αPD-1 and AZD0156, either alone or in combination. Control mice received isotype control antibody and vehicle. Tumor growth curves of a single experiment (**M**; mouse number = 5–8; see also Supplementary Fig. S7B) and relative AUC analysis (**N**). Representative IHC staining (**O**) and relative quantification (**P**) of CD8 T cells in the tumor core (*n*_tr_ = FoV = 10 in **P**). **Q** and **R,** Overall survival of mice injected with TC-1 + myoMLF (**Q**) or MC38 + myoMCF (**R**) and treated as indicated (mouse number = 7–8 for both experiments; Mantel–Cox log-rank test is shown; see also Supplementary Fig. S7C and S7D). Scale bars, 200 μm. A homoscedastic Student *t* test is used throughout the figure and is relative to the control unless otherwise highlighted. ns, nonsignificant; *, *P* ≤ 0.05; **, *P* ≤ 0.01; ***, *P* ≤ 0.001; ****, *P* ≤ 0.0001.

We have shown previously that myoCAF confer immunotherapy resistance by excluding CD8 T cells from tumors (14). Therefore, we tested whether ATM inhibition could reverse this effect. Analysis of myoCAF-rich TC-1 and MC38 tumors by IHC showed that ATM inhibition (fibroblast shRNA knockdown and AZD0156) resulted in a significant relocation of CD8 T cells from the tumor periphery to the tumor core (Fig. 6A–D, G, and H; Supplementary Fig. S6A and S6B). CD4 T-cell infiltration was unaffected (Supplementary Fig. S6C–H). Flow cytometry analysis showed that ATM inhibition did not affect CD8 T-cell phenotype/function in myoCAF-rich tumors (Fig. 6E and F).

We next investigated whether the effect of ATM inhibition on intratumoral CD8 T cells could potentiate the response to immunotherapy. First, we tested a vaccine model using HPV E6/E7-expressing TC-1 cells, combining AZD0156 with a DNA vaccine directed against E7 (RAH; ref. 34); we have shown previously that myoCAF-rich TC-1 tumors are resistant to the vaccine response (14). Mice bearing CAF-rich TC-1 tumors were treated with AZD0156 or vaccine monotherapy, vaccine/drug combination, or control. While AZD0156 monotherapy was effective in reducing intratumoral myoCAF accumulation (Supplementary Fig. S6I and S6J), the drug/vaccine combination significantly increased CD8 T-cell infiltration and reduced tumor volume compared with the single treatments (Fig. 6I–L and Supplementary Fig. S7A). AZD0156 was also tested in combination with αPD-1 in mice bearing myoCAF-rich MC38 tumors, a model where, similarly, myoCAF confer resistance to anti–PD-1 therapy (14). We found again that the combination of αPD-1 with AZD0156 produced the most significant CD8 T-cell influx and reduction in tumor growth (Fig. 6M–P and Supplementary Figs. S6K, S6L, and S7B). Survival experiments performed over a longer time period showed that combining ATM inhibition with immunotherapy increased the overall survival compared with single treatments alone in both tumor models (Fig. 6Q and R; Supplementary Fig. S7C and D). When tumors relapsed upon αPD1 ± AZD0156, a second treatment with the combination was administered, resulting in significantly fewer myoCAF and increased CD8 T cells (Supplementary Fig. S7E–S7G). Notably, despite the high levels of CD8 T cells in these tumors, mice showed only a nonsignificant trend for increased survival (Supplementary Fig. S7H–S7O). Overall, these data suggest that ATM inhibition is effective at promoting the infiltration of CD8 T cells into myoCAF-rich tumors and can be used to potentiate the early response to immunotherapy.

## Discussion

MyoCAF are associated with poor prognosis in many cancers and have been shown to promote tumor immune evasion. However, effective targeting of this cell population has not yet been accomplished (16, 19), in part confounded by the fact that CAF remain a poorly defined, heterogeneous cell population (7, 17). Recent single-cell transcriptomic analyses have identified novel CAF phenotypes, including iCAF and antigen-presenting CAF (apCAF), although it is not yet clear how widely these CAF subgroups are found in different cancers or how they function (40). Most research has focused on myoCAF. These are present to a greater or lesser extent in most types of solid tumors, usually abutting tumor cells, and depositing a desmoplastic stroma rich in collagens, fibronectin, and proteoglycans that has been shown to “trap” T cells and limit T-cell access to the tumor core (14, 41, 42). Notably, expression of TGFβ-associated myoCAF ECM genes is one of the strongest predictors of immunotherapy failure, highlighting the ability of myoCAF to create an immunosuppressive tumor microenvironment (8–12). Here, we show that the activation of ATM plays a central role in promoting and maintaining the myoCAF phenotype and that ATM can be effectively targeted to “normalize” myoCAF, overcome myoCAF-mediated immune evasion, and potentiate response to immunotherapy.

ATM, along with ATR and DNA-PKcs, is principally known for its role as an apical kinase in the DDR pathway. However, ATM signaling has been shown to also regulate other cell functions, including glucose metabolism (43), cell homeostasis (44), and multiple differentiation processes (45). We found that TGFβ1-induced myofibroblast differentiation results in the activation of ATM and CHK2 in the presence of significantly increased (albeit low) DNA breaks. The localized pattern of pH2AX and pATM staining in the nuclei of cells treated with TGFβ1 confirmed the presence of DNA damage in the form of DSBs (30), consistent with TGFβ/SMAD signaling, promoting ATM activation and downstream signaling as a result of direct DNA damage (25–27, 46). In keeping with this, we found that inhibiting MRN complex/DNA damage activation of ATM by targeting Mre11 inhibits TGFβ1 induction of the kinase.

In breast CAF, ATM has also been shown to be activated by oxidative stress in the absence of DNA damage, suggesting potential activation of the ATM dimer (47). Here, we found that ATM activation results from both oxidation and DNA damage and that both are dependent on ROS generated by NOX4. We established that NOX4 (but not Mre11) activates ATM as a dimer, indicating a ROS-driven oxidation of the kinase independent of monomer activation by the MRN complex. Consistent with NOX4 being reported to promote DDR activation in several cell types (25, 27), here we demonstrate that NOX4 is the source of ROS promoting ATM dimer formation during the myofibroblast/myoCAF differentiation process. This likely explains the lack of KAP1 activation typically observed during oxidative stress activation of ATM (31) and proposes a model whereby there exists a parallel activation of ATM by DNA damage and oxidative stress, both induced by NOX4.

We found that ATM was activated in myoCAF isolated *ex vivo* from NSCLC and HNSCC, also detected by multiplexed immunochemistry in the SMA-positive stroma of the same tumors. GSEA also showed enrichment of ATM/DDR-related genes in the myofibroblastic stroma of ovarian, esophageal, liver, and colorectal tumors, consistent with proteomic analysis of esophageal and breast CAF performed elsewhere (48, 49). In support of its role in myoCAF differentiation, ATM activation has additionally been reported to contribute to various fibrotic conditions, including systemic sclerosis (50), renal (51), and hepatocellular fibrosis (52), all myofibroblast-dependent processes, suggesting, overall, that the association of ATM signaling and myofibroblast/myoCAF phenotypes holds true across different tissues and disease pathogenesis. Continued ATM activation in myoCAF isolated *ex vivo* suggested that it also plays a role in maintaining the myoCAF phenotype. We found that ATM inhibition normalized myoCAF, downregulating SMA and other myoCAF markers while promoting an iCAF phenotype, consistent with recent studies that have highlighted plasticity in CAF populations, showing that CAF subgroups are not fixed in the state of terminal differentiation (37, 53).

We also investigated whether DDR upstream or downstream components of the ATM pathway can regulate myofibroblastic CAF phenotype and found that Mre11 and CHK2 are also involved in myofibroblast differentiation. This contrasts with previous work that identified CHK2 as a repressor of breast myofibroblastic CAF phenotype (54), although it is consistent with the increased DDR gene expression and ATM activation observed in breast CAF by several other groups (47, 49)

ATM signaling involvement in myoCAF differentiation is in keeping with our previous data showing that DNA damaging agents, including irradiation, can induce a contractile, SMA-positive myofibroblastic-like phenotype through an ATM-dependent mechanism (20). Additionally, we have shown that continuous TGFβ1 treatment of fibroblasts over time promotes senescence (35), which is commonly triggered by DNA damage. It appears therefore that myofibroblast activation and senescence may be two linked stress responses, perhaps with TGFβ1 induction of ATM signaling and the myofibroblast phenotype, followed by later senescence forming part of the same differentiation program.

The recognition that myoCAF provide a major resistance mechanism to immunotherapy has renewed interest in CAF targeting as an immunotherapy adjunct, and different therapeutic approaches have been suggested. TGFβ signaling is the major pathway regulating myoCAF differentiation, and different groups have shown that coadministration of anti-TGFβ with anti–PD-1/PD-L1 antibodies significantly improves response in preclinical tumor models (8, 13). TGFβ blockage has been shown to reduce the formation of myoCAF and promote the formation of an “interferon-licensed” CAF subpopulation with increased immunomodulatory properties, resulting in greater anti–PD-1 efficacy (53). Inhibiting TGFβ, however, can be problematic; this pleiotropic cytokine has multiple roles in normal physiology as well as tumor-suppressive effects, and its targeting has led to on-target cardiac toxicities in preclinical studies, as well as the development of cutaneous tumors in human trials (18, 55). Inhibiting the mechanisms by which CAF exclude T cells from tumors is an alternative approach to overcome immunotherapy resistance and perhaps the most attractive strategy to do that is to ‘normalize’ myoCAF, particularly since studies have suggested that certain CAF/fibroblast phenotypes may be tumor-suppressive (56). We previously identified the ROS-producing enzyme NOX4 as a key regulator of the myoCAF phenotype (14, 28). Inhibition of NOX4 using the small-molecule inhibitor GKT137831/Setanaxib suppresses myoCAF differentiation and overcomes myoCAF-mediated immunotherapy resistance (14). Here, we found that ATM is activated by NOX4 and plays a similar role in regulating and maintaining myoCAF differentiation.

Drugs inhibiting ATM and other components of the DDR are generally effective in tumors with specific preexisting DNA-repair defects or are used in combination with platinum compounds or ionizing radiation where they are associated with significant toxicity (57). As monotherapy, ATM inhibitors are well tolerated, with dose escalation phase I clinical studies with the ATM inhibitors AZD0156 or AZD1390 not yet reporting to be associated with adverse effects. However, ATM inhibitor monotherapy is generally therapeutically ineffective in conventional preclinical tumor models (58, 59), where the stromal component is typically absent (14). Nonetheless, recent studies have shown that ATM inhibitors can promote response to checkpoint immunotherapy through cGAS/STING signaling in tumor cells, resulting in an enhanced interferon I response and increased immunogenicity (58). Notably, we found that ATM inhibition did not affect control (CAF-low) tumors comprising TC-1 and MC38 cells alone, but its effect was limited to myoCAF-rich tumors, where it reversed myoCAF differentiation and potentiated immunotherapy response. Whether this latter effect is modulated through the downregulation of ECM proteins or altered expression of inflammatory cytokines remains to be determined (41, 58), but our data suggest that the use of ATM inhibitors can be expanded for stromal targeting.

In summary, we identify ATM activation as a novel, targetable pathway regulating myoCAF differentiation. Most types of solid tumors have a myoCAF-rich subgroup; given the major role of myoCAF in suppressing response to anti–PD-1/PD-L1 checkpoint inhibitors (8, 13), targeting this pathway as part of combination immunotherapy could have significant therapeutic benefit.

## Supplementary Material

Supplementary Data 2 list of primer sequences, antibody details and primary fibroblasts used

Supplementary Data 3 GSEA between different tumour stromal datasets from GEO and DDR & myoCAF genesets

Supplementary Data 4 Significant DDR genesets (from Leading-Edge analysis in GSEA) used to make the DDR_TGFB_signature geneset

Supplementary Data 5 Non-redundant list of the leading-edge genes and ToppFun pathway analysis from the genesets in Suppl. File 4 used to make the DDR-TGFB_signature geneset

Supplementary Figures and Legends supplementary figures and legends

Macros for image quantification macros for image quantifications

## References

[1] Fearon DT . Immune-suppressing cellular elements of the tumor microenvironment. Ann Rev Cancer Biol 2017;1:241–55.

[2] Kalluri R . The biology and function of fibroblasts in cancer. Nat Rev Cancer 2016;16:582–98. 27550820 10.1038/nrc.2016.73

[3] Hinz B , Phan SH, Thannickal VJ, Prunotto M, DesmouliÃ¨re A, Varga J, . Recent developments in myofibroblast biology: paradigms for connective tissue remodeling. Am J Pathol 2012;180:1340–55. 22387320 10.1016/j.ajpath.2012.02.004 PMC3640252

[4] Marsh D , Suchak K, Moutasim KA, Vallath S, Hopper C, Jerjes W, . Stromal features are predictive of disease mortality in oral cancer patients. J Pathol 2011;223:470–81. 21294121 10.1002/path.2830

[5] Underwood TJ , Hayden AL, Derouet M, Garcia E, Noble F, White MJ, . Cancer-associated fibroblasts predict poor outcome and promote periostin-dependent invasion in oesophageal adenocarcinoma. J Pathol 2015;235:466–77. 25345775 10.1002/path.4467 PMC4312957

[6] Yamashita M , Ogawa T, Zhang X, Hanamura N, Kashikura Y, Takamura M, . Role of stromal myofibroblasts in invasive breast cancer: stromal expression of alpha-smooth muscle actin correlates with worse clinical outcome. Breast Cancer 2012;19:170–6. 20978953 10.1007/s12282-010-0234-5

[7] Hanahan D , Coussens LM. Accessories to the crime: functions of cells recruited to the tumor microenvironment. Cancer Cell 2012;21:309–22. 22439926 10.1016/j.ccr.2012.02.022

[8] Mariathasan S , Turley SJ, Nickles D, Castiglioni A, Yuen K, Wang Y, . TGFbeta attenuates tumour response to PD-L1 blockade by contributing to exclusion of T cells. Nature 2018;554:544–8. 29443960 10.1038/nature25501 PMC6028240

[9] Hugo W , Zaretsky JM, Sun L, Song C, Moreno BH, Hu-Lieskovan S, . Genomic and transcriptomic features of response to anti-pd-1 therapy in metastatic melanoma. Cell 2017;168:542. 10.1016/j.cell.2017.01.010 28129544

[10] Chakravarthy A , Khan L, Bensler NP, Bose P, De Carvalho DD. TGF-beta-associated extracellular matrix genes link cancer-associated fibroblasts to immune evasion and immunotherapy failure. Nat Commun 2018;9:4692. 30410077 10.1038/s41467-018-06654-8 PMC6224529

[11] Dominguez CX , Müller S, Keerthivasan S, Koeppen H, Hung J, Gierke S, . Single-cell RNA sequencing reveals stromal evolution into lrrc15(+) myofibroblasts as a determinant of patient response to cancer immunotherapy. Cancer Discov 2020;10:232–53. 31699795 10.1158/2159-8290.CD-19-0644

[12] Kieffer Y , Hocine HR, Gentric G, Pelon F, Bernard C, Bourachot B, . Single-cell analysis reveals fibroblast clusters linked to immunotherapy resistance in cancer. Cancer Discov 2020;10:1330–51. 32434947 10.1158/2159-8290.CD-19-1384

[13] Tauriello DVF , Palomo-Ponce S, Stork D, Berenguer-Llergo A, Badia-Ramentol J, Iglesias M, . TGFbeta drives immune evasion in genetically reconstituted colon cancer metastasis. Nature 2018;554:538–43. 29443964 10.1038/nature25492

[14] Ford K , Hanley CJ, Mellone M, Szyndralewiez C, Heitz F, Wiesel P, . NOX4 inhibition potentiates immunotherapy by overcoming cancer-associated fibroblast-mediated CD8 T-cell exclusion from tumors. Cancer Res 2020. 10.1158/0008-5472.CAN-19-3158 PMC7611230 32122909

[15] Togo S , Polanska U, Horimoto Y, Orimo A. Carcinoma-associated fibroblasts are a promising therapeutic target. Cancers (Basel) 2013;5:149–69. 24216702 10.3390/cancers5010149 PMC3730310

[16] Narra K , Mullins SR, Lee H-O, Strzemkowski-Brun B, Magalong K, Christiansen VJ, . Phase II trial of single-agent Val-boroPro (talabostat) inhibiting fibroblast activation protein in patients with metastatic colorectal cancer. Cancer Biol Ther 2007;6:1691–9. 18032930 10.4161/cbt.6.11.4874

[17] Sugimoto H , Mundel TM, Kieran MW, Kalluri R. Identification of fibroblast heterogeneity in the tumor microenvironment. Cancer Biol Ther 2006;5:1640–6. 17106243 10.4161/cbt.5.12.3354

[18] Morris JC , Tan AR, Olencki TE, Shapiro GI, Dezube BJ, Reiss M, . Phase I study of GC1008 (fresolimumab): a human anti-transforming growth factor-beta (TGFbeta) monoclonal antibody in patients with advanced malignant melanoma or renal cell carcinoma. PLoS One 2014;9:e90353. 24618589 10.1371/journal.pone.0090353 PMC3949712

[19] Catenacci DVT , Junttila MR, Karrison T, Bahary N, Horiba MN, Nattam SR, . Randomized phase Ib/II study of gemcitabine plus placebo or vismodegib, a hedgehog pathway inhibitor, in patients with metastatic pancreatic cancer. J Clin Oncol 2015;33:4284–92. 26527777 10.1200/JCO.2015.62.8719 PMC4678179

[20] Mellone M , Hanley CJ, Thirdborough S, Mellows T, Garcia E, Woo J, . Induction of fibroblast senescence generates a non-fibrogenic myofibroblast phenotype that differentially impacts on cancer prognosis. Aging (Albany NY) 2016;9:114–32. 27992856 10.18632/aging.101127 PMC5310659

[21] Jackson SP , Bartek J., The DNA-damage response in human biology and disease. Nature 2009;461:1071–8. 19847258 10.1038/nature08467 PMC2906700

[22] Blackford AN , Jackson SP. ATM, ATR, and DNA-PK: the trinity at the heart of the DNA damage response. Mol Cell 2017;66:801–17. 28622525 10.1016/j.molcel.2017.05.015

[23] Barcellos-Hoff MH , Cucinotta FA. New tricks for an old fox: impact of TGFbeta on the DNA damage response and genomic stability. Sci Signal 2014;7:re5. 25185158 10.1126/scisignal.2005474

[24] Kirshner J , Jobling MF, Pajares MJ, Ravani SA, Glick AB, Lavin MJ, . Inhibition of transforming growth factor-beta1 signaling attenuates ataxia telangiectasia mutated activity in response to genotoxic stress. Cancer Res 2006;66:10861–9. 17090522 10.1158/0008-5472.CAN-06-2565

[25] Hubackova S , Kucerova A, Michlits G, Kyjacova L, Reinis M, Korolov O, . IFNgamma induces oxidative stress, DNA damage and tumor cell senescence via TGFbeta/SMAD signaling-dependent induction of Nox4 and suppression of ANT2. Oncogene 2016;35:1236–49. 25982278 10.1038/onc.2015.162

[26] Dickey JS , Baird BJ, Redon CE, Sokolov MV, Sedelnikova OA, Bonner WM. Intercellular communication of cellular stress monitored by gamma-H2AX induction. Carcinogenesis 2009;30:1686–95. 19651821 10.1093/carcin/bgp192 PMC2757548

[27] Razdan N , Vasilopoulos T, Herbig U., Telomere dysfunction promotes transdifferentiation of human fibroblasts into myofibroblasts. Aging Cell 2018;17:e12838. 30244523 10.1111/acel.12838 PMC6260909

[28] Hanley CJ , Mellone M, Ford K, Thirdborough SM, Mellows T, Frampton SJ, . Targeting the myofibroblastic cancer-associated fibroblast phenotype through inhibition of NOX4. J Natl Cancer Inst 2018;110. 10.1093/jnci/djx121 PMC5903651 28922779

[29] Paull TT . Mechanisms of ATM Activation. Annu Rev Biochem 2015;84:711–38. 25580527 10.1146/annurev-biochem-060614-034335

[30] Bakkenist CJ , Kastan MB. DNA damage activates ATM through intermolecular autophosphorylation and dimer dissociation. Nature 2003;421:499–506. 12556884 10.1038/nature01368

[31] Guo Z , Kozlov S, Lavin MF, Person MD, Paull TT. ATM activation by oxidative stress. Science 2010;330:517–21. 20966255 10.1126/science.1192912

[32] Bauer JA , Trask DK, Kumar B, Los G, Castro J, Shin-Jung Lee J, . Reversal of cisplatin resistance with a BH3 mimetic, (-)-gossypol, in head and neck cancer cells: role of wild-type p53 and Bcl-xL. Mol Cancer Ther 2005;4:1096–104. 16020667 10.1158/1535-7163.MCT-05-0081

[33] Serrander L , Cartier L, Bedard K, Banfi B, Lardy B, Plastre O, . NOX4 activity is determined by mRNA levels and reveals a unique pattern of ROS generation. Biochem J 2007;406:105–14. 17501721 10.1042/BJ20061903 PMC1948990

[34] Allen A , Wang C, Caproni LJ, Sugiyarto G, Harden E, Douglas LR, . Linear doggybone DNA vaccine induces similar immunological responses to conventional plasmid DNA independently of immune recognition by TLR9 in a pre-clinical model. Cancer Immunol Immunother 2018;67:627–38. 29330557 10.1007/s00262-017-2111-y PMC5860099

[35] Hassona Y , Cirillo N, Lim KP, Herman A, Mellone M, Thomas GJ, . Progression of genotype-specific oral cancer leads to senescence of cancer-associated fibroblasts and is mediated by oxidative stress and TGF-beta. Carcinogenesis 2013;34:1286–95. 23358854 10.1093/carcin/bgt035

[36] Guo Z , Deshpande R, Paull TT. ATM activation in the presence of oxidative stress. Cell Cycle 2010;9:4805–11. 21150274 10.4161/cc.9.24.14323 PMC3047807

[37] Biffi G , Oni TE, Spielman B, Hao Y, Elyada E, Park Y, . IL1-induced JAK/STAT signaling is antagonized by TGFbeta to shape CAF heterogeneity in pancreatic ductal adenocarcinoma. Cancer Discov 2019;9:282–301. 30366930 10.1158/2159-8290.CD-18-0710 PMC6368881

[38] Anderson VE , Walton MI, Eve PD, Boxall KJ, Antoni L, Caldwell JJ, . CCT241533 is a potent and selective inhibitor of CHK2 that potentiates the cytotoxicity of PARP inhibitors. Cancer Res 2011;71:463–72. 21239475 10.1158/0008-5472.CAN-10-1252 PMC4948722

[39] Bai G , Hock TD, Logsdon N, Zhou Y, Thannickal VJ. A far-upstream AP-1/Smad binding box regulates human NOX4 promoter activation by transforming growth factor-beta. Gene 2014;540:62–7. 24560583 10.1016/j.gene.2014.02.026 PMC4009368

[40] Elyada E , Bolisetty M, Laise P, Flynn WF, Courtois ET, Burkhart RA, . Cross-species single-cell analysis of pancreatic ductal adenocarcinoma reveals antigen-presenting cancer-associated fibroblasts. Cancer Discov 2019;9:1102–23. 31197017 10.1158/2159-8290.CD-19-0094 PMC6727976

[41] Salmon H©L¨N , Franciszkiewicz K, Damotte D, Dieu-Nosjean M-C, Validire P, Trautmann A, . Matrix architecture defines the preferential localization and migration of T cells into the stroma of human lung tumors. J Clin Invest 2012;122:899–910. 22293174 10.1172/JCI45817 PMC3287213

[42] Evanko SP , Potter-Perigo S, Bollyky PL, Nepom GT, Wight TN. Hyaluronan and versican in the control of human T-lymphocyte adhesion and migration. Matrix Biol 2012;31:90–100. 22155153 10.1016/j.matbio.2011.10.004 PMC3288568

[43] Armata HL , Golebiowski D, Jung DY, Ko HJ, Kim JK, Sluss HK. Requirement of the ATM/p53 tumor suppressor pathway for glucose homeostasis. Mol Cell Biol 2010;30:5787–94. 20956556 10.1128/MCB.00347-10 PMC3004274

[44] Lee J-H , Mand MR, Kao C-H, Zhou Y, Ryu SW, Richards AL, . ATM directs DNA damage responses and proteostasis via genetically separable pathways. Sci Signal 2018;11. 10.1126/scisignal.aan5598 PMC5898228 29317520

[45] Sherman MH , Bassing CH, Teitell MA. Regulation of cell differentiation by the DNA damage response. Trends Cell Biol 2011;21:312–9. 21354798 10.1016/j.tcb.2011.01.004 PMC3089693

[46] Legrand AJ , Poletto M, Pankova D, Clementi E, Moore J, Castro-Giner F, . Persistent DNA strand breaks induce a CAF-like phenotype in normal fibroblasts. Oncotarget 2018;9:13666–81. 29568385 10.18632/oncotarget.24446 PMC5862606

[47] Tang S , Hou Y, Zhang H, Tu G, Yang L, Sun Y, . Oxidized ATM promotes abnormal proliferation of breast CAFs through maintaining intracellular redox homeostasis and activating the PI3K-AKT, MEK-ERK, and Wnt-beta-catenin signaling pathways. Cell Cycle 2015;14:1908–24. 25970706 10.1080/15384101.2015.1041685 PMC4615140

[48] Manousopoulou A , Hayden A, Mellone M, Garay-Baquero DJ, White CH, Noble F, . Quantitative proteomic profiling of primary cancer-associated fibroblasts in oesophageal adenocarcinoma. Br J Cancer 2018;118:1200–7. 29593339 10.1038/s41416-018-0042-9 PMC5943522

[49] Peng Q , Zhao L, Hou Y, Sun Y, Wang L, Luo H, . Biological characteristics and genetic heterogeneity between carcinoma-associated fibroblasts and their paired normal fibroblasts in human breast cancer. PLoS One 2013;8:e60321. 23577100 10.1371/journal.pone.0060321 PMC3618271

[50] Svegliati S , Marrone G, Pezone A, Spadoni T, Grieco A, Moroncini G, . Oxidative DNA damage induces the ATM-mediated transcriptional suppression of the Wnt inhibitor WIF-1 in systemic sclerosis and fibrosis. Sci Signal 2014;7:ra84. 25185156 10.1126/scisignal.2004592

[51] Daugherity EK , Balmus G, Al Saei A, Moore ES, Abi Abdallah D, Rogers AB, . The DNA damage checkpoint protein ATM promotes hepatocellular apoptosis and fibrosis in a mouse model of non-alcoholic fatty liver disease. Cell Cycle 2012;11:1918–28. 22544329 10.4161/cc.20259 PMC3359121

[52] Overstreet JM , Samarakoon R, Cardon-Grau D, Goldschmeding R, Higgins PJ. Tumor suppressor ataxia telangiectasia mutated functions downstream of TGF-beta1 in orchestrating profibrotic responses. FASEB J 2015;29:1258–68. 25480384 10.1096/fj.14-262527 PMC4396616

[53] Grauel AL , Nguyen B, Ruddy D, Laszewski T, Schwartz S, Chang J, . TGFbeta-blockade uncovers stromal plasticity in tumors by revealing the existence of a subset of interferon-licensed fibroblasts. Nat Commun 2020;11:6315. 33298926 10.1038/s41467-020-19920-5 PMC7725805

[54] Al-Rakan MA , Hendrayani SF, Aboussekhra A. CHEK2 represses breast stromal fibroblasts and their paracrine tumor-promoting effects through suppressing SDF-1 and IL-6. BMC Cancer 2016;16:575. 27484185 10.1186/s12885-016-2614-5 PMC4970236

[55] Anderton MJ , Mellor HR, Bell A, Sadler C, Pass M, Powell S, . Induction of heart valve lesions by small-molecule ALK5 inhibitors. Toxicol Pathol 2011;39:916–24. 21859884 10.1177/0192623311416259

[56] Helms E , Onate MK, Sherman MH. Fibroblast heterogeneity in the pancreatic tumor microenvironment. Cancer Discov 2020;10:648–56. 32014869 10.1158/2159-8290.CD-19-1353 PMC8261791

[57] Pilié PG , Tang C, Mills GB, Yap TA. State-of-the-art strategies for targeting the DNA damage response in cancer. Nat Rev Clin Oncol 2019;16:81–104. 30356138 10.1038/s41571-018-0114-z PMC8327299

[58] Hu M , Zhou M, Bao X, Pan D, Jiao M, Liu X, . ATM inhibition enhances cancer immunotherapy by promoting mtDNA leakage and cGAS/STING activation. J Clin Invest 2021;131. 10.1172/JCI139333 PMC7843232 33290271

[59] Durant ST , Zheng L, Wang Y, Chen K, Zhang L, Zhang T, . The brain-penetrant clinical ATM inhibitor AZD1390 radiosensitizes and improves survival of preclinical brain tumor models. Sci Adv 2018;4:eaat1719. 29938225 10.1126/sciadv.aat1719 PMC6010333

