# Supplementary material for: ATM Regulates Differentiation of Myofibroblastic Cancer-Associated Fibroblasts and Can Be Targeted to Overcome Immunotherapy Resistance
Source: Cancer Res. 2022 Nov 10;82(24):4571–85. doi: 10.1158/0008-5472.CAN-22-0435 (PMC9755965; doi:10.1158/0008-5472.CAN-22-0435)
Supplement: Supplementary Figures and Legends [file can-22-0435_supplementary_figures_and_legends_suppsfl1-sfl7.pdf]

Fig. S1

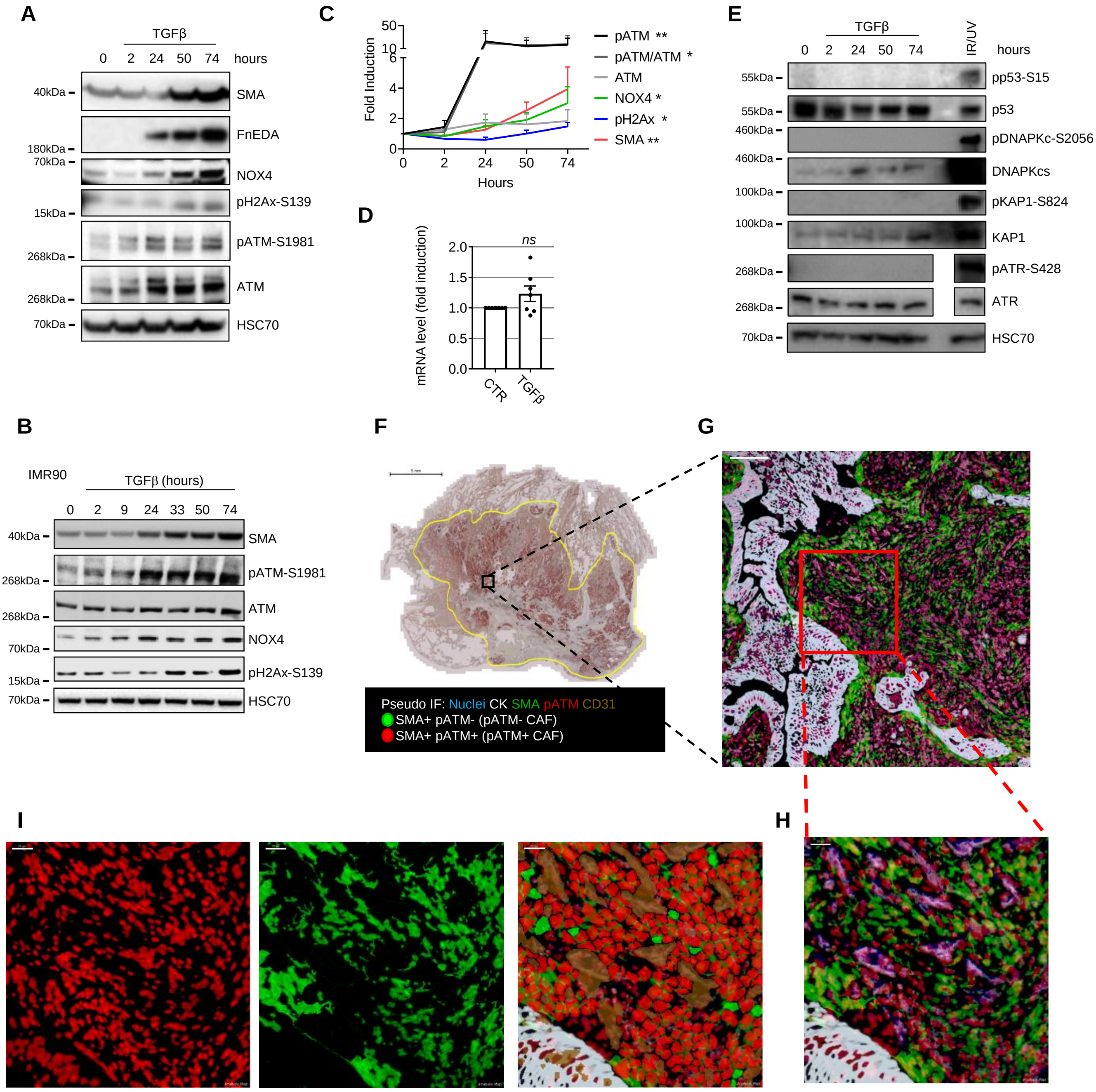
A
TGFβ
0 2 24 50 74 hours
40kDa
SMA
FnEDA
180kDa
70kDa
NOX4
15kDa
pH2Ax-S139
pATM-S1981
268kDa
ATM
268kDa
70kDa
HSC70
C
Fold Induction
Hours
pATM **
pATM/ATM *
ATM
NOX4 *
pH2Ax *
SMA **
D
mRNA level (fold induction)
ns
CTR
TGFβ
E
TGFβ
0 2 24 50 74
IR/UV
hours
55kDa
pp53-S15
55kDa
p53
460kDa
pDNAPKc-S2056
460kDa
DNAPKcs
100kDa
pKAP1-S824
100kDa
KAP1
268kDa
pATR-S428
268kDa
ATR
70kDa
HSC70
B
IMR90
TGFβ (hours)
0 2 9 24 33 50 74
40kDa
SMA
268kDa
pATM-S1981
268kDa
ATM
268kDa
NOX4
70kDa
pH2Ax-S139
15kDa
70kDa
HSC70
F
Pseudo IF: Nuclei CK SMA pATM CD31
SMA+ pATM- (pATM- CAF)
SMA+ pATM+ (pATM+ CAF)
G
I
H

Supplementary Figure 1: ATM activation in myofibroblasts. A,B) Western blotting of HFFF2 (**A**) and IMR90 (**B**) fibroblasts treated with TGF-β1 over time. **C**) Quantification of the proteins shown in the Western blotting from Supplementary Fig. 1A,B, Fig. 1A (n_{br}=3; Kruskall-Wallis statistical test is used). **D**) Q-RT-PCR of HFFF2 treated with TGF-β1 for 72 hours (n_{tr}=2-3) showing ATM mRNA is not modulated by TGF-β1. **E**) Western blotting of HFFF2 treated with TGF-β1 over time. The single cropped bands for ATR or pATR belong to the same gel and image of the rest of the blot. **F-I**) Representative image of NSCLC MxIHC; bright field image of cytokeratin staining (scale bar=5mm **F**); pseudo-colored images (scale bar=100μm **G**; scale bar=20μm **H-I**); single-stained and merged pseudo-colored images with the cell regions used for the quantification highlighted in red or green for pATM or SMA positivity respectively (subtracted CD31 staining shown in brown). Heteroscedastic Student's t-test is used in the figure and refers to the control unless otherwise stated.

Fig. S2

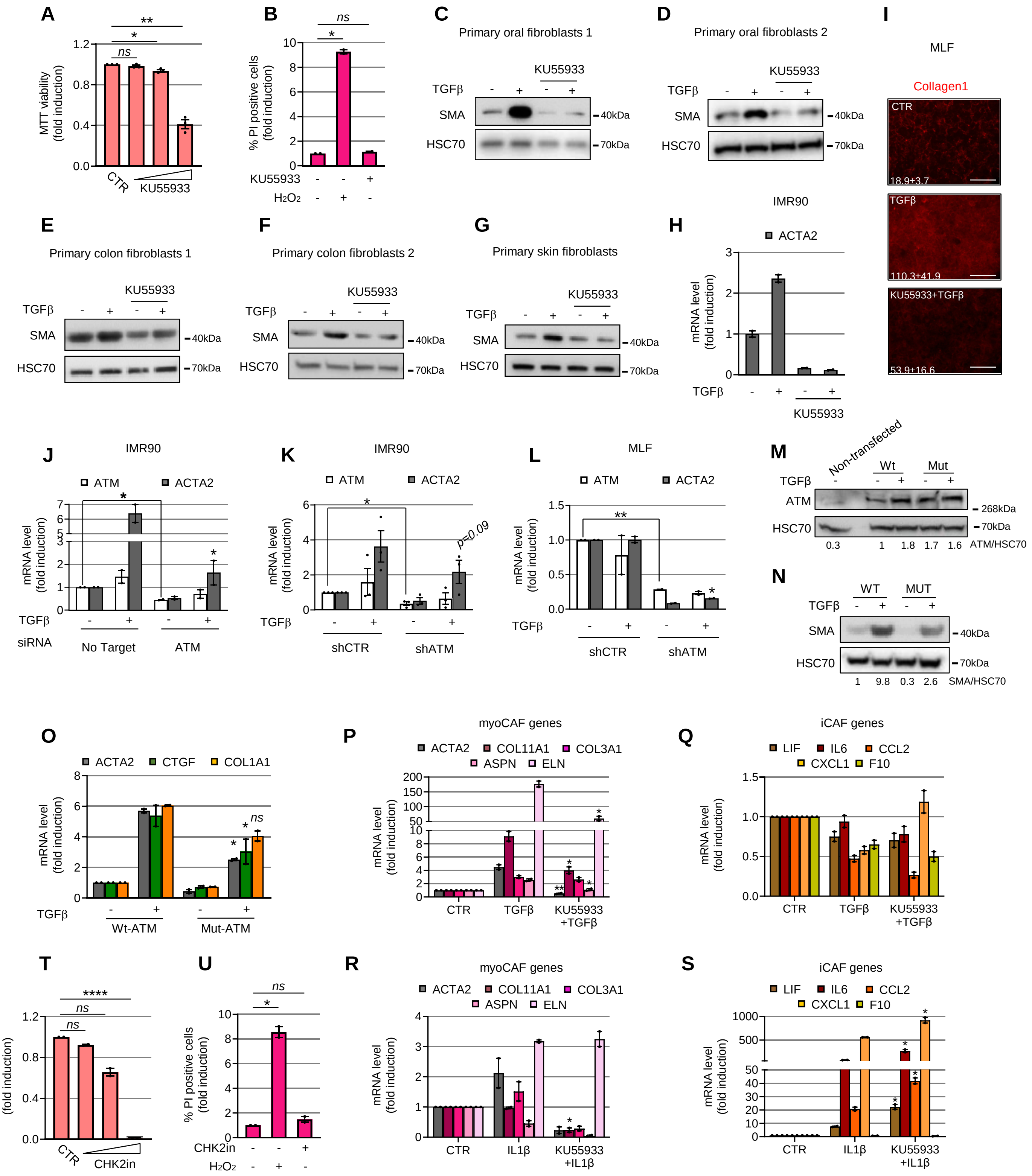

A
MTT viability (fold induction)
CTR
KU55933
B
% PI positive cells (fold induction)
KU55933
H2O2
C
Primary oral fibroblasts 1
D
Primary oral fibroblasts 2
TGFβ
SMA
HSC70
40kDa
70kDa
E
Primary colon fibroblasts 1
F
Primary colon fibroblasts 2
G
Primary skin fibroblasts
H
IMR90
ACTA2
mRNA level (fold induction)
I
MLF
Collagen1
CTR
18.9±3.7
110.3±41.9
KU55933+TGFβ
53.9±16.6
J
ATM
siRNA
No Target
K
shCTR
shATM
p=0.09
L
M
Non-transfected
Wt
Mut
268kDa
ATM/HSC70
N
WT
MUT
SMA/HSC70
O
CTGF
COL1A1
Wt-ATM
Mut-ATM
P
myoCAF genes
COL11A1
COL3A1
ASPN
ELN
KU55933 +TGFβ
Q
iCAF genes
LIF
IL6
CCL2
CXCL1
F10
T
CHK2in
U
R
IL1β
KU55933 +IL1β
S

Supplementary Fig. 2: ATM inhibition suppresses myofibroblast differentiation. A) MTT assay of HFFF2 treated with 13.3µM, 20µM or 40µM KU55933 for 72 hours (n_{tr}=2). **B**) PI assay of HFFF2 treated with 13.3µM KU55933 for 72 hours or with H_2O_2 (as positive control; n_{tr}=2). **C-G**) Western blotting of human primary oral, colon and skin fibroblasts isolated from healthy donors treated with KU55933±TGF-β1 for 72 hours (densitometry analysis shown in Fig. 2C). **H**) Representative Q-RT-PCR of IMR90 fibroblasts treated as in C-G (n_{br}=1; n_{tr}=2 shown in the plot). **I**) Representative immunofluorescence staining of collagen1 and relative quantification in mouse lung fibroblasts (MLF) treated with KU55933 ±TGF-β1 for one week (scale bar=500µm). **J-L**) Q-RT-PCR of fibroblasts transfected with ATM siRNA (**J**; n_{tr}=2), or stably expressing ATM shRNA (**K,L**) and treated as in C-G (n_{tr}=3). **M-O**) Western blotting/quantification (**M,N**) and Q-RT-PCR (**O**; n_{tr}=3) of HFFF2 stably expressing wild type (wt) or mutant ATM (mut) and treated as in C-G. **P-S**) Q-RT-PCR of HFFF2 treated with KU55933 ± TGF-β1 or 10ng/ml IL-1β for 72 hours **(**n_{tr}=3). **T**) MTT assay of HFFF2 treated with 1.5µM, 3µM, 10µM CHK2 inhibitor CCT241533 (n_{tr}=2) for 72 hours. **U**) PI assay of HFFF2 treated with 1.5µM CHK2 inhibitor for 72 hours or H_2O_2 as positive control (n_{tr}=2). Heteroscedastic Student T-test is used in the figure and it refers to TGF-β1-treated samples unless otherwise highlighted.

Fig. S3

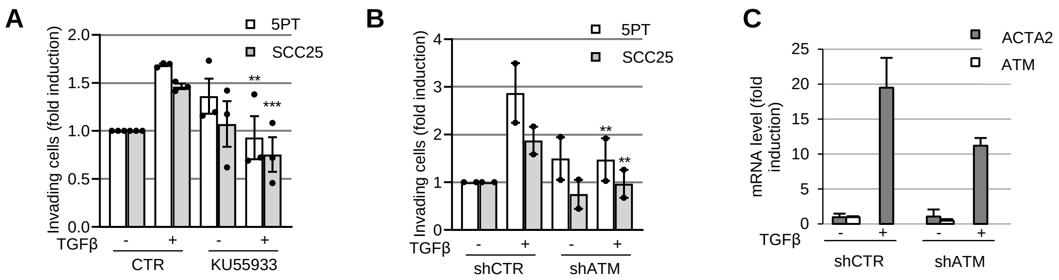

Supplementary Fig. 3: ATM inhibition reverses the myofibroblast CAF phenotype and inhibits function. A,B) Transwell invasion assays of 5PT or SCC25 cells towards conditioned media generated from HFFF2 treated with TGF-β1 +/- KU55933 for 7 days (n_{tr}=2-4 for 5PT and SCC25). **C**) Representative Q-RT-PCR of HFFF2 in B showing ATM and ACTA2 mRNA expression prior to the invasion assay (n_{br}=1, n_{tr}=3; Standard deviation is shown). Two-way Anova is shown in the figure and refers to the TGF-β1 treated samples.

Fig. S4

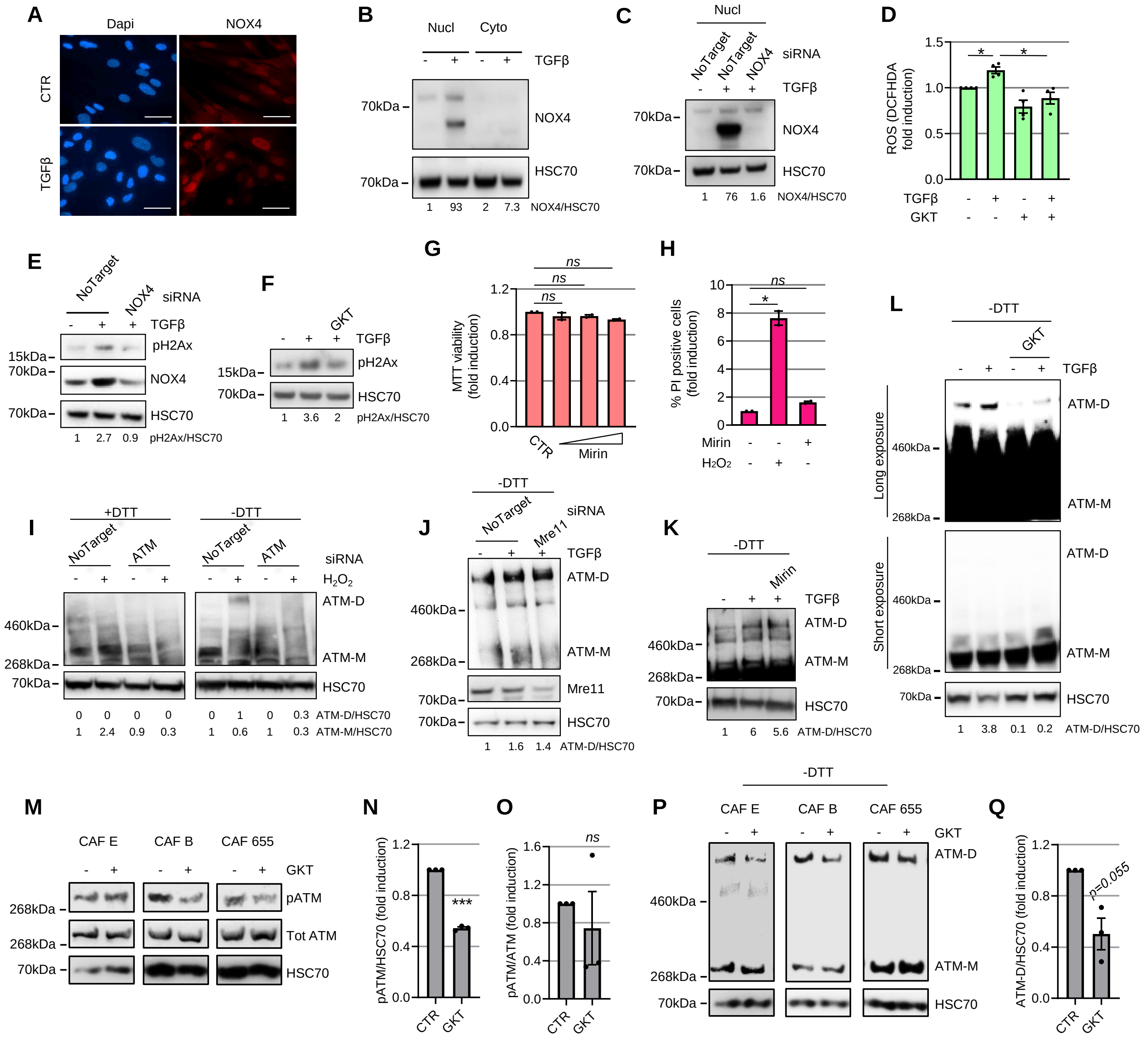
A
Dapi
NOX4
CTR
TGFβ
B
Nucl
Cyto
TGFβ
70kDa
NOX4
HSC70
1 93 2 7.3 NOX4/HSC70
C
Nucl
NoTarget
NoTarget
NOX4
siRNA
TGFβ
70kDa
NOX4
HSC70
1 76 1.6 NOX4/HSC70
D
ROS (DCFHDA fold induction)
TGFβ
GKT
E
NoTarget
NOX4
siRNA
TGFβ
15kDa
70kDa
pH2Ax
NOX4
HSC70
1 2.7 0.9 pH2Ax/HSC70
F
GKT
TGFβ
pH2Ax
HSC70
1 3.6 2 pH2Ax/HSC70
G
MTT viability (fold induction)
ns
CTR
Mirin
H
% PI positive cells (fold induction)
ns
Mirin
H2O2
L
-DTT
GKT
TGFβ
ATM-D
ATM-M
460kDa
268kDa
Long exposure
Short exposure
HSC70
1 3.8 0.1 0.2 ATM-D/HSC70
I
+DTT
-DTT
NoTarget
ATM
siRNA
H2O2
ATM-D
ATM-M
HSC70
0 0 0 0 0 1 0 0.3 ATM-D/HSC70
1 2.4 0.9 0.3 1 0.6 1 0.3 ATM-M/HSC70
J
-DTT
NoTarget
Mre11
siRNA
TGFβ
ATM-D
ATM-M
Mre11
HSC70
1 1.6 1.4 ATM-D/HSC70
K
-DTT
Mirin
TGFβ
ATM-D
ATM-M
HSC70
1 6 5.6 ATM-D/HSC70
M
CAF E
CAF B
CAF 655
GKT
pATM
Tot ATM
HSC70
N
pATM/HSC70 (fold induction)
***
CTR
GKT
O
pATM/ATM (fold induction)
ns
CTR
GKT
P
-DTT
CAF E
CAF B
CAF 655
GKT
ATM-D
ATM-M
HSC70
Q
ATM-D/HSC70 (fold induction)
p=0.055
CTR
GKT

Supplementary Fig. 4: TGFβ activates ATM via NOX4-driven DNA damage/MRN complex and oxidation. A) Representative immunofluorescence staining of HFFF2 treated with TGF-β1 for 24 hours showing nuclear NOX4. Dapi used as nuclear counter-stain (scale bar=50μm). **B,C**) Western blotting/quantification of nuclear/cytoplasm (**A**) or nuclear (**B**) extracts of HFFF2 transfected with NOX4 siRNA and treated for 48 hours with TGF-β1 showing nuclear NOX4. **D-F**) HFFF2 were treated for 48 hours with TGF-β1. ROS assay showing the DCFH-DA mean fluorescence of HFFF2 treated with GKT137831 (40μM; n_{tr}=2-3; **D**); Western blotting/quantification of HFFF2 transfected with NOX4 siRNA (**E**) or treated with GKT137831 (**F**). **G**) MTT assay of HFFF2 treated with 8μM, 40μM or 200μM Mirin for 72 hours (n_{tr}=2). **H**) PI assay of HFFF2 treated with 40μM Mirin for 72 hours or with H_2O_2 (as positive control; n_{tr}=2). **I**) Western blotting/quantification of HFFF2 transfected with the indicated siRNAs and treated for 60 minutes with 2mM H_2O_2 (left and right gel run in reducing and non-reducing conditions respectively; Dithiothreitol=DTT). **J-L**) Non-reducing Western blotting/quantification (-DTT) of HFFF2 treated for 48 hours with TGF-β1 and as indicated. **M-Q**) Western blotting [(reducing (**M**) and non-reducing (**P**)] and their quantification (**N,O,Q**) of nuclear extracts from two HNSCC CAF and one NSCLC CAF (E, B & 655 respectively) treated daily with GKT137831 for 72 hours. Heteroscedastic Student's T-test is used throughout the figure.

Fig. S5

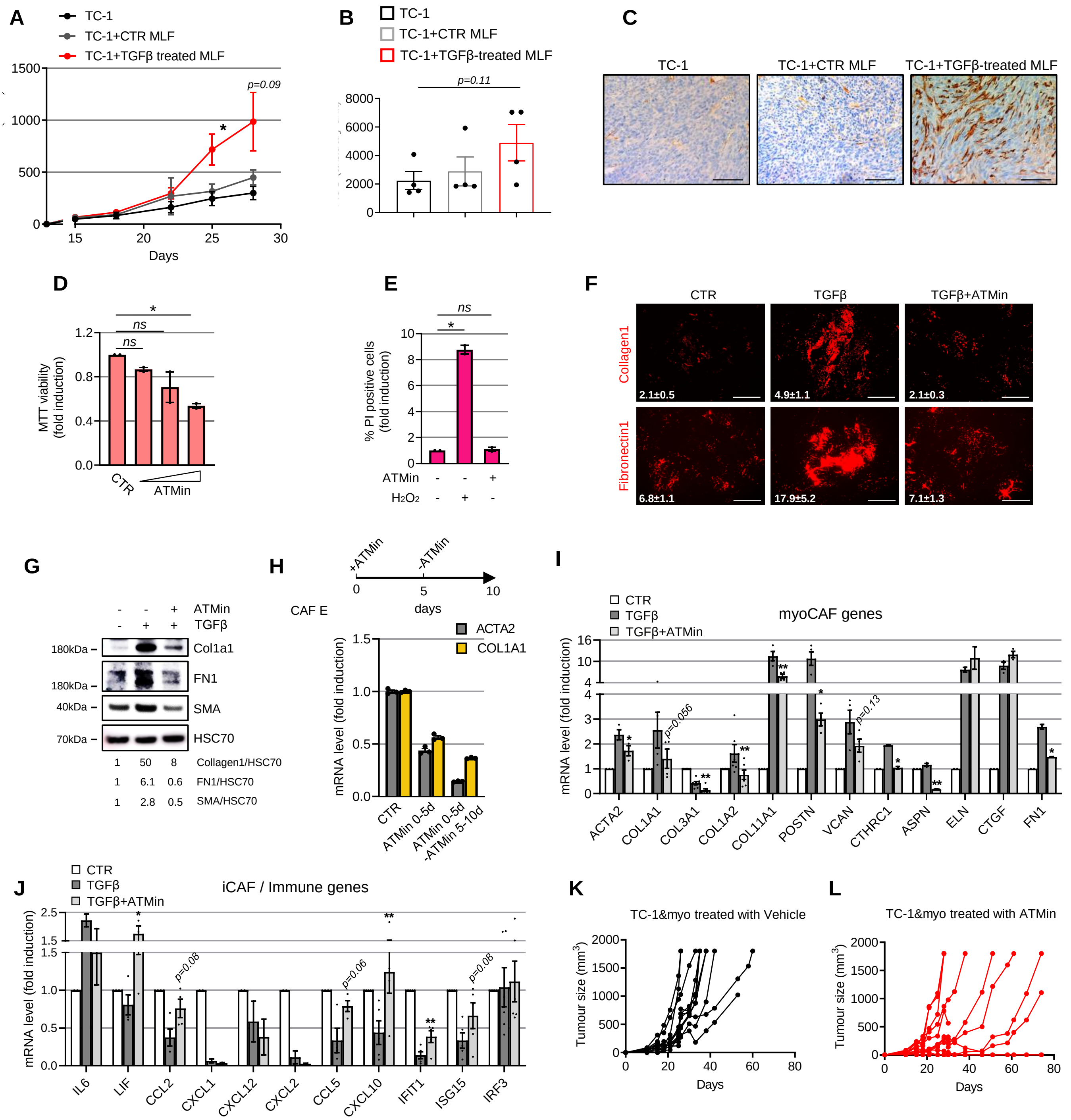

A
TC-1
TC-1+CTR MLF
TC-1+TGFβ treated MLF
p=0.09
*
1500
1000
500
0
15
20
25
30
Days
B
TC-1
TC-1+CTR MLF
TC-1+TGFβ-treated MLF
p=0.11
8000
6000
4000
2000
0
C
TC-1
TC-1+CTR MLF
TC-1+TGFβ-treated MLF
D
*
ns
ns
1.2
0.8
0.4
0.0
MTT viability (fold induction)
CTR
ATMin
E
ns
*
10
8
6
4
2
0
% PI positive cells (fold induction)
ATMin - - +
H2O2 - + -
F
CTR
TGFβ
TGFβ+ATMin
Collagen1
Fibronectin1
2.1±0.5
4.9±1.1
2.1±0.3
6.8±1.1
17.9±5.2
7.1±1.3
G
- - + ATMin
- + + TGFβ
180kDa
Col1a1
180kDa
FN1
40kDa
SMA
70kDa
HSC70
1 50 8 Collagen1/HSC70
1 6.1 0.6 FN1/HSC70
1 2.8 0.5 SMA/HSC70
H
+ATMin
-ATMin
0
5
10
days
CAF E
ACTA2
COL1A1
1.5
1.0
0.5
0.0
mRNA level (fold induction)
CTR
ATMin 0-5d
ATMin 0-5d -ATMin 5-10d
I
CTR
TGFβ
TGFβ+ATMin
myoCAF genes
16
10
4
4
3
2
1
0
mRNA level (fold induction)
p=0.056
p=0.13
ACTA2
COL1A1
COL3A1
COL1A2
COL11A1
POSTN
VCAN
CTHRC1
ASPN
ELN
CTGF
FN1
J
CTR
TGFβ
TGFβ+ATMin
iCAF / Immune genes
2.5
1.5
1.5
1.0
0.5
0.0
mRNA level (fold induction)
p=0.08
p=0.06
p=0.08
IL6
LIF
CCL2
CXCL1
CXCL12
CXCL2
CCL5
CXCL10
IFIT1
ISG15
IRF3
K
TC-1&myo treated with Vehicle
2000
1500
1000
500
0
Tumour size (mm3)
0
20
40
60
80
Days
L
TC-1&myo treated with ATMin
2000
1500
1000
500
0
Tumour size (mm3)
0
20
40
60
80
Days

Supplementary Fig. 5: Targeting ATM in myofibroblasts reduces myoCAF gene expression, their intratumoral accumulation and inhibits tumor growth. A-C) Mice were injected with TC-1+/- untreated (CTR) or TGF-β1-treated MLF (myoMLF; mouse number=4); tumor growth curves (**A**; the Homoscedastic Student's T-tests shown are relative to the comparison between the tumor size means in the latest two time points of TC-1 vs TC-1+TGFβ1-treated MLF samples) AUC analysis (**B**) representative SMA IHC stainings (**C**) of the mouse tumors from a single experiment (scale bar=100μm). **D**) MTT assay of HFFF2 treated with TGF-β1 for 72 hours +/-AZD0156 (0.5μM, 1.25μM, 2.5μM; n_{tr}=4). **E**) PI assay of HFFF2 treated for 72 hours with 0.5μM AZD0156 or with H_2O_2 (as positive control; n_{tr}=2). **F**) Representative immunofluorescent staining for Collagen1 and Fibronectin and relative quantification of the mean in MLF treated for 7 days with TGF-β1+/-AZD0156 (0.5μM; scale bar=500μm; FoV=10). **G**) Western blotting/quantification of MLF treated with TGF-β1+/-AZD0156 (0.5μM) for 72 hours. **H**) Q-RT-PCR of CAF E treated with AZD0156 for 5 days and then left them grow for further 5 days without AZD0156 (n_{tr}=3 shown in the plot). **I,J**) Q-RT-PCR of MLF treated with TGF-β1+/-AZD0156 (0.5μM) for a week (n_{br}=2-7; n_{tr}=3). **K,L**) Individual mouse tumor volume measurements relative to Fig. 5O. Mice were injected with TC-1+myoMLF and either treated with vehicle (mouse number=11; **K**) or treated with AZD0156 (mouse number=12; **L**). Heteroscedastic Student's T-test is used throughout the figure unless differently highlighted.

Fig. S6

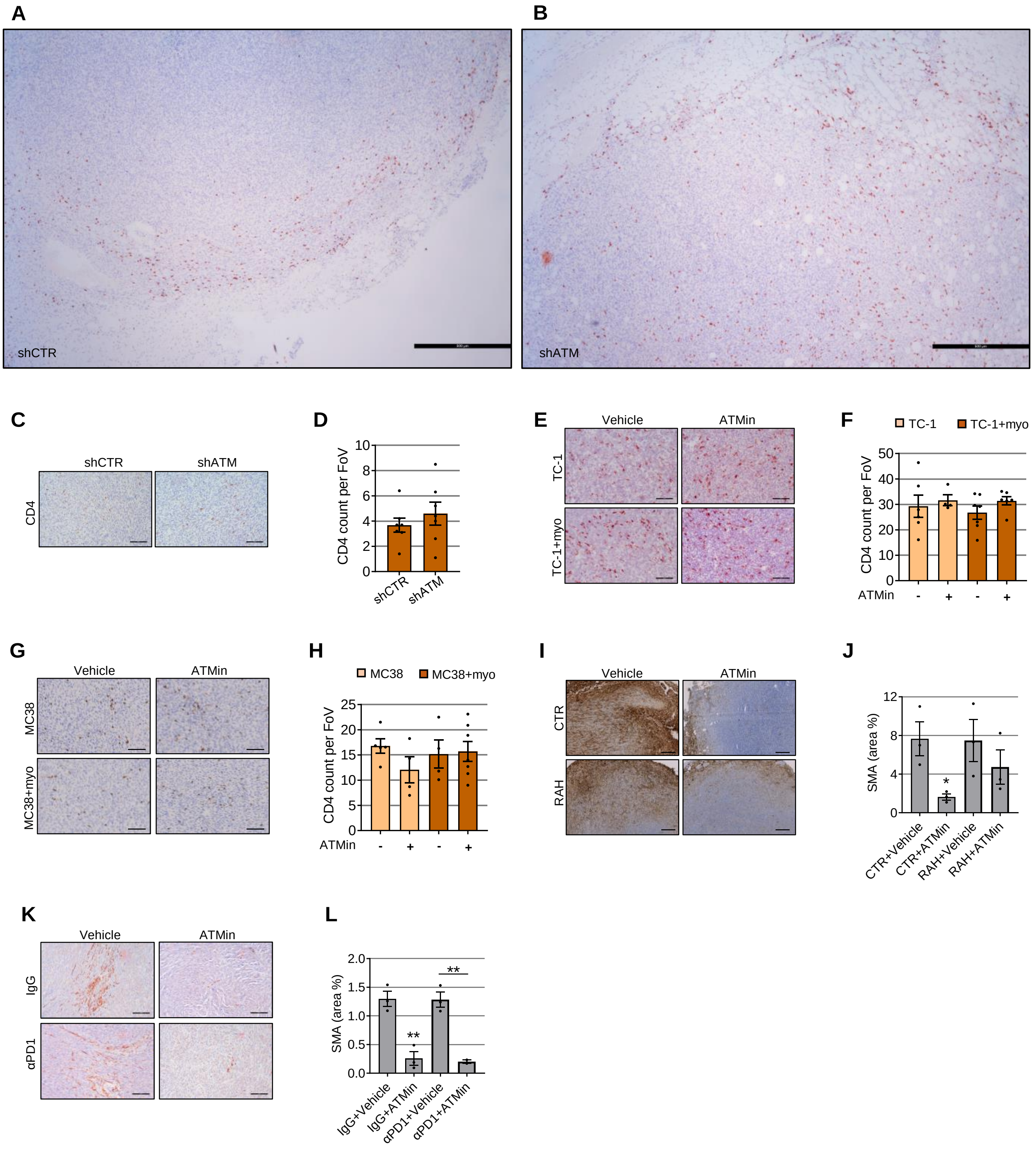

A
B
shCTR
shATM
C
D
E
F
shCTR
shATM
CD4
CD4 count per FoV
Vehicle
ATMin
TC-1
TC-1+myo
CD4 count per FoV
ATMin
G
H
I
J
Vehicle
ATMin
MC38
MC38+myo
CD4 count per FoV
ATMin
CTR
RAH
SMA (area %)
CTR+Vehicle
CTR+ATMin
RAH+Vehicle
RAH+ATMin
K
L
Vehicle
ATMin
IgG
αPD1
SMA (area %)
IgG+Vehicle
IgG+ATMin
αPD1+Vehicle
αPD1+ATMin

Supplementary Fig. 6: Targeting ATM in myofibroblasts promotes tumor CD8 T-cell infiltration. A,B) Representative images of CD8 IHC staining (at low magnification) in TC-1 myo-rich tumors (in **Fig. 6A,5G**) with shCTR or shATM knockdown in myoMLF (scale bars=500µm). **C,D**) Representative images of IHC staining (**C**) and relative quantification (**D**) of CD4 T-cells in the core of tumors described in Fig. 5G (n_{tr}=FoV=5-10). **E,F**) Representative IHC staining (**E**) and relative quantification (**F**) of CD4 T-cells in the core of tumors described in Fig. 5K (n_{tr}=FoV=5-10). **G,H**) Representative IHC staining (**G**) and relative quantification (**H**) of CD4 T-cells in the core of the tumors described in Fig. 5P (n_{tr}=FoV=10).). **I,J**) Representative images of SMA IHC staining (**I**) and relative quantification (**J**) of the mouse tumors described in Fig. 6I (ntr=FoV=3). **K,L**) Representative images of SMA IHC staining (**K**) and relative quantification (**L**) of the mouse tumors described in Fig. 6M (ntr=FoV=3). All scale bars=200µm and the homoscedastic Student T-test is relative to control unless differently highlighted.

Fig. S7

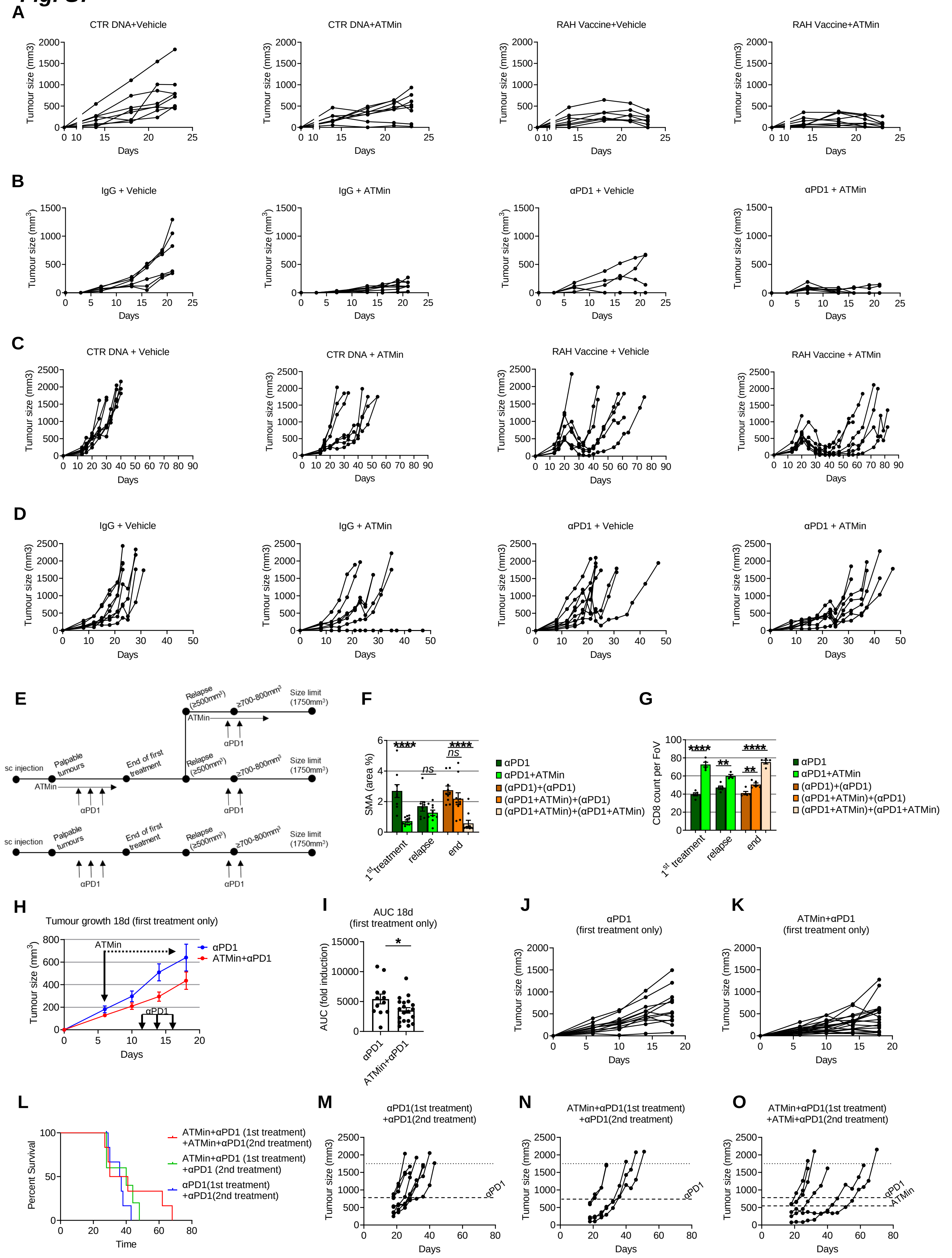

A
CTR DNA+Vehicle
CTR DNA+ATMin
RAH Vaccine+Vehicle
RAH Vaccine+ATMin
Tumour size (mm3)
Days
B
IgG + Vehicle
IgG + ATMin
αPD1 + Vehicle
αPD1 + ATMin
C
CTR DNA + Vehicle
CTR DNA + ATMin
RAH Vaccine + Vehicle
RAH Vaccine + ATMin
D
IgG + Vehicle
IgG + ATMin
αPD1 + Vehicle
αPD1 + ATMin
E
sc injection
Palpable tumours
ATMin
αPD1
End of first treatment
Relapse (≥500mm³)
≥700-800mm³
Size limit (1750mm³)
F
SMA (area %)
αPD1
αPD1+ATMin
(αPD1)+(αPD1)
(αPD1+ATMin)+(αPD1)
(αPD1+ATMin)+(αPD1+ATMin)
1st treatment
relapse
end
ns
G
CD8 count per FoV
H
Tumour growth 18d (first treatment only)
ATMin+αPD1
I
AUC 18d (first treatment only)
AUC (fold induction)
J
αPD1 (first treatment only)
K
ATMin+αPD1 (first treatment only)
L
Percent Survival
Time
ATMin+αPD1 (1st treatment) +ATMin+αPD1(2nd treatment)
ATMin+αPD1 (1st treatment) +αPD1 (2nd treatment)
αPD1(1st treatment) +αPD1(2nd treatment)
M
αPD1(1st treatment) +αPD1(2nd treatment)
N
ATMin+αPD1(1st treatment) +αPD1(2nd treatment)
O
ATMin+αPD1(1st treatment) +ATMi+αPD1(2nd treatment)

Supplementary Fig. 7: Targeting ATM in myofibroblasts potentiates immunotherapy. A) Individual mouse tumor volume measurements relative to Fig. 6I. **B**) Individual mouse tumor volume measurements relative to Fig. 6M. **C**) Individual mouse tumor volume measurements relative Fig. 6Q. **D**) Individual mouse tumor volume measurements relative to Fig. 6R. **E-O**) Mice were injected with MC38+myoMCF and treated with αPD-1+/-AZD0156 for 10 days (first treatment; day 6-16). The second treatment with AZD0156 was given at relapse (size≥500mm^3) followed by two doses of αPD-1 (size≥700/800mm^3)(schematic of the experiment shown in **E**). SMA (**F;** FoV=n_{tr}=8-11) and CD8 (**G;** FoV=n_{tr}=4-5) quantification of IHC staining (not shown) of single tumors from mice after the first treatment, at relapse or at the end of the experiment [at tumor size limit=1750mm^3 (top dotted line in **M-O**)]. The tumor growth curves (**H**), the AUC plot (**i**) and the single tumor growth curves (**J,K**) are relative to the earliest part of the experiment when all the mice received the first treatment (homoscedastic Student T-test is shown in **I**). Overall survival analysis (**L**) and single tumor growth curves (**M-O**) are relative to all the mice which relapsed after the first treatment (tumor size≥500mm^3) and received the second treatment (mouse number=5-7; Mantel-Cox log-rank test is not significant).
